# Lactococcal phage–host profiling through binding studies between cell wall polysaccharide types and Skunavirus receptor-binding proteins

**DOI:** 10.1099/mgen.0.001395

**Published:** 2025-04-28

**Authors:** Kelsey White, Giovanni Eraclio, Brian McDonnell, Gabriele Andrea Lugli, Tadhg Crowley, Marco Ventura, Federica Volonté, Christian Cambillau, Fabio Dal Bello, Jennifer Mahony, Douwe van Sinderen

**Affiliations:** 1 School of Microbiology, University College Cork, Cork, T12 Y337, Ireland; 2 APC Microbiome Ireland, University College Cork, Cork, T12 YT20, Ireland; 3 Sacco Srl, Via Manzoni 29/A, 22071, Cadorago (Co), Italy; 4 Laboratory of Probiogenomics, Department of Chemistry, Life Sciences, and Environmental Sustainability, University of Parma, 12 - I 43121 Parma, Italy; 5 Flow Cytometry Platform, APC Microbiome Ireland, University College Cork, Cork, Ireland; 6 Laboratoire d’Ingénierie des Systèmes Macromoléculaires (LISM), Institut de Microbiologie, Bioénergies et Biotechnologie (IMM), Aix-Marseille Université – CNRS, UMR 7255, Marseille, France

**Keywords:** bacteriophage, carbohydrate binding, dairy fermentation, *Lactococcus cremoris*, *Lactococcus lactis*, receptor-binding protein

## Abstract

Dairy fermentations using mesophilic starter cultures rely on the activity of specific lactic acid bacteria (LAB) such as *Lactococcus lactis* and *Lactococcus cremoris* for the acidification of milk. This biotechnological process can be affected by bacteriophage infection of LAB starter strains, which may result in delayed or even failed fermentations. Most studied lactococcal phages commence infection with the binding of a tail-associated receptor-binding protein (RBP) to a host cell surface-exposed cell wall polysaccharide (CWPS). In the present study, phage prevalence and diversity in whey samples originating from fermentations performed in various European countries employing undefined mesophilic starter cultures were investigated using phageome analysis. The range of *Skunavirus* RBP genotypes present in the phageomes and associated RBP-CWPS binding abilities were evaluated, resulting in the refinement and expansion of the *Skunavirus* RBP grouping system and the identification of several heretofore unknown *Skunavirus* RBP (sub)groups. These findings substantially expand our knowledge on lactococcal *Skunavirus* RBP diversity and their binding specificity towards CWPS receptor structures, thereby improving the predictability of fermentation outcomes and robustness of starter culture rotations and blends.

**Impact Statement**

*Lactococcus lactis* and *Lactococcus cremoris* strains are extensively used in mesophilic dairy fermentation systems. Bacteriophage infection of starter bacteria is one of the most significant and persistent threats to successful dairy fermentation processes. Lactococcal strains produce cell wall polysaccharides, which represent the saccharidic receptor of many lactococcal phages and therefore can be linked to their phage susceptibility profiles. In this study, the specific phage–host interactions occurring between the strictly lytic lactococcal *Skunavirus* phages and their cognate hosts were investigated. This was achieved through a thorough examination of the diversity and binding specificity of *Skunavirus* receptor-binding proteins (RBPs) present in a number of dairy fermentation-derived whey phageomes. This analysis subsequently led to the refinement and expansion of the previous *Skunavirus* RBP grouping system. This work provides significant insights into the potential susceptibility of lactococcal strains to specific skunaviruses (based on their *cwps* and *rbp* genotypes), which may allow for more effective and deliberate starter culture rotation strategies, thereby reducing the impact and risk of future phage attacks.

## Data Summary

Raw virome sequences are available at the National Center for Biotechnology Information (NCBI) Sequence Read Archive (https://www.ncbi.nlm.nih.gov/sra) under the BioProject accession number PRJNA1176342 (individual BioSample accession numbers are included in Table 1). Receptor-binding protein (RBP) sequences retrieved from virome assemblies are available on GenBank under accession numbers PQ639347–PQ639427 (individual accession numbers for each RBP are listed in Table S1, available in the online Supplementary Material). Predicted structures are accessible on Zenodo (https://zenodo.org/doi/10.5281/zenodo.11221961).

**Table 1.** Sample information and BioSample accession numbers for sequenced whey virome samples

Sample code	Date	Country	BioSample accession no.
T1	06/2019	Spain	SAMN44397675
BH1	07/2021		SAMN44397676
BR	01/2022		SAMN44397677
BT	04/2022		SAMN44397678
AD1	12/2019	Austria	SAMN44397679
AF3	02/2020	France	SAMN44397680
AF9	02/2020		SAMN44397681
BQ	11/2022		SAMN44397682
BA6	10/2020	Poland	SAMN44397683
BB4	10/2020		SAMN44397684
BZ	10/2022		SAMN44397685
AO	05/2020	Sweden	SAMN44397686
V1	06/2019	Denmark	SAMN44397687
V2	06/2019		SAMN44397688
AE3	02/2020		SAMN44397689
AI3	03/2020		SAMN44397690
AM2	03/2020		SAMN44397691
AN3	04/2020		SAMN44397692
AN4	04/2020		SAMN44397693
AV6	09/2020		SAMN44397694
BC9	02/2021		SAMN44397695
BD	03/2021		SAMN44397696
BE9	03/2021		SAMN44397697
BV	07/2022		SAMN44397698
CA	11/2022		SAMN44397699
CD	01/2023		SAMN44397700
BP	10/2021	Czechia	SAMN44397701

## Introduction

Milk fermentations involving mesophilic starter cultures are typically reliant on the activity of *Lactococcus cremoris* and/or *Lactococcus lactis* for the manufacture of a variety of fermented dairy products. The primary role of these micro-organisms in fermentations is to produce lactic acid, which leads to a pH reduction and consequent coagulation of milk and curd formation. Bacterial dairy starter cultures are broadly classified as either defined (a small number of strains in a known combination) or undefined (an unknown number of strains in an undetermined relative abundance) [1]. There are advantages and disadvantages associated with either of these starter culture formulations, particularly with respect to bacteriophage (or phage) susceptibility, which represents one of the most significant challenges in the fermentation industry. Phage infection can retard or even arrest fermentation, which can lead to substantial economic losses [2]. Therefore, phages associated with dairy fermentations (particularly lactococcal phages) have been extensively studied in recent decades [3,8].

Lactococcal phages are classified into ten distinct taxonomic groups: *Skunavirus* (previously 936), P335, *Ceduovirus* (previously c2), *Whiteheadvirus* (previously 1358), *Questintvirus* (previously Q54), *Teubervirus* (previously P087), *Fremauxvirus* (previously 1706), *Audreyjarvisvirus* (previously 949), P034 and *Chopinvirus* (previously KSY1) [9]. Skunaviruses, ceduoviruses and P335 phages are, by a substantial margin, the most commonly encountered phages in commercial, high-intensity dairy fermentations [5,10]. Skunaviruses and ceduoviruses are both strictly virulent, whereas P335 phages may either be virulent or temperate in nature [11,12]. Despite efforts to limit and control phage proliferation in dairy fermentation factories, lactococcal phages remain a persistent issue [12,14]. Phage populations in dairy fermentation environments, therefore, require constant monitoring and investigation. Historically, culture-based methods were applied to detect, enumerate and characterize phages [15]. However, these methods typically identify the most dominant and propagatable phages present, thereby not comprehensively accounting for the overall phage population, in particular low abundance phages or phages that propagate poorly (or not at all) under laboratory conditions. The recent surge in metagenomic (and phageomics, representing the investigation of all phage-derived genetic material within a sample) platforms and analysis tools allows a broader, in-depth understanding of phage diversity, prevalence and abundance in a number of food fermentation processes [16,19]. Traditional culture-based methods are biassed towards the most dominant and propagatable portions of the viral fraction, whereas phageome analysis provides insights into the prevalence, diversity, abundance and community dynamics occurring in these fermentation environments. Although virome analyses have been performed for various ecological niches, there is a lack of assembly methods that are optimized towards viral rather than bacterial genome assembly, leading to an underrepresentation of the true viral population [20,21]. This is particularly problematic when resolving phage genomes, which tend to be highly mosaic compared to bacterial genomes and often harbour repeat regions. Attempting to remedy this, the Phables software has recently been developed to identify phage-like components from assembly graphs and to more accurately and completely resolve phage genome assemblies [22]. Nevertheless, for functional characterization of phages, it is still necessary to identify the corresponding sensitive host of a particular phage, suggesting that a combination of cultivation- and DNA-based analyses is most suitable to provide a comprehensive view of the viral population [23].

Phage infection typically initiates with the recognition and reversible binding of a phage-encoded receptor binding protein (RBP) to a specific receptor present on a host’s cell surface. The RBP of skunaviruses is located at the distal end of the phage tail as part of a large heteropolymeric protein complex [24]. The RBP recognizes and binds to a specific cell wall polysaccharide (CWPS), (part of) which is exposed on the host’s cell surface [25,28]. A lactococcal CWPS is composed of two distinct parts: a peptidoglycan-embedded rhamnan and a surface-exposed poly- or oligosaccharidic side chain (referred henceforth as the CWPS side chain), the latter believed to act as the primary phage receptor [29,30]. The CWPS side chain of lactococcal strains is chemically diverse and this is underpinned by the observed genetic variability within the 3′ region of lactococcal *cwps* gene clusters, which is responsible for its biosynthesis. Currently, 4 *cwps* types, termed *cwps* A through to *cwps* D, and 11 C subtypes (designated C_1_ through to C_11_) are described, although it is expected that the true diversity of these gene clusters and their associated structures far exceeds those identified to date [25,27]. The compositional and structural diversity of lactococcal CWPS side chains partly underpins the typically narrow host range of skunaviruses [27], while it also explains the associated diversity in the *Skunavirus* RBPs [26,28, 31].

All *Skunavirus* RBPs possess a conserved structure consisting of three modules, i.e. the so-called ‘head’, ‘neck’ and ‘shoulder’ domains [32]. The C-terminally located RBP head domain elicits carbohydrate binding activity, which facilitates specific binding to the host’s saccharidic receptor, i.e. the CWPS side chain as mentioned above. *Skunavirus* RBPs have been classified into five groups (groups I–V) based on aa sequence similarity levels of their C-terminal (encoding the RBP head domain) sequences [28,31]. Recently, a phylogenetic analysis of over 200 *Skunavirus* RBP sequences from an industrial phage collection indicated the potential presence of additional RBP groups [3]. Bacteria often incorporate mutations or alterations to host receptors to evade phage infection [33]. Phages, in turn, may modify their RBPs, typically by acquiring mutations or through genetic exchange, highlighting the constant co-evolution of host-encoded receptors and phage-encoded RBPs. The need for continuous and comprehensive examination of binding specificity correlations between *Skunavirus* RBPs and host CWPSs is evident, therefore [34,35].

In the current study, viromes (all virus-like particles and corresponding to deoxynucleic acids within a sample) from 27 whey samples collected from several dairy fermentation factories across Europe employing mesophilic, undefined starter cultures were extracted and sequenced. The corresponding phageomes (assembled phage-like genetic components) were analysed to investigate the diversity of RBP genotypes of skunaviruses in these factories, facilitating an expanded and refined *Skunavirus* RBP classification system. The emergent *Skunavirus* RBP (sub)groups identified in the analysed whey phageomes were examined through structural predictions using AlphaFold2 [36,37] and *in vitro* binding assays. A previously described phage RBP-activated cell sorting (PhRACS) approach was used to identify specific RBP-host interactions [23]. The PhRACS method was recently established as an effective method of retrieving low-abundance phage–host combinations from complex samples. This is achieved through specific labelling of culturable bacterial hosts with phage RBPs coupled to a fluorescent tag, thereby facilitating isolation of such an RBP-bound host by flow cytometry. As a result of the co-evolutionary dynamics between bacteria and phages, the structural diversity observed for both lactococcal host cell wall receptors and *Skunavirus* RBPs appears to be a key contributor to the perpetual battle between bacteria and their infecting phages.

## Methods

### Cultivation, propagation and storage of lactococcal strains and bacteriophages

Bacterial strains used in this study are presented in Table 2. Lactococcal strains were routinely grown at 30 °C in M17 broth supplemented with 0.5% lactose (LM17; Millipore Sigma Aldrich, Gillingham, UK). Phages were propagated (initially from single plaques, then using 1% of phage lysates after initial plaque propagation) in the same medium supplemented with 10 mM CaCl_2_ and added at the point of host strain inoculation (1% of fresh overnight culture), after which the infected culture was incubated overnight at 30 °C. The resulting phage lysates were filtered (0.45 µM; Sarstedt, Nümbrecht, Germany) and stored at 4 °C.

**Table 2.** Bacterial strains used in this study

Lactococcal strain*	CWPS type	GenBank accession no.	Source/reference
UC509.9	A	CP003157	UCC
**IL1403**	B	AE005176	[71]
MG1363	C₁	AM406671	[72]
3107	C_2_	CP031538	[73]
SK11	C₃	CP000425	[74]
W34	C₄	CP032430	UCC
1196	C₄	CP032148	UCC
**IO-1**	C₅	AP012281	[75]
**UC06**	C_5_	CP015902	UCC
A76	C₆	CP003132	[76]
**Tome1F**	C_7_	NZ_JAHIBU000000000	[68]
Peco9D	C_7_	NZ_JAHIBV000000000	[68]
RMN8G	C_9_	NZ_JAHIBS000000000	[68]
**Tempeh6L**	C_10_	NZ_JAHIBT000000000	[68]
**TolaII67**	C_11_	JAHIBR000000000	[68]
**184**	D	CP015895	UCC

*L. lactis strains are bolded, and the remaining strains are *L. cremoris*.

### Virome extraction and sequencing

Dairy-derived whey samples (Table 1) were acquired from factories where diminished milk acidification activity of starter cultures was observed. The obtained whey samples were stored at −20 °C prior to processing. Viral particles were isolated from whey samples using a previously described viral extraction method [38], as follows: samples were thawed on ice and 5 to 10 ml of whey (depending on the originally received volume) was centrifuged at 300 ***g*** for 5 min at 4 °C to remove any large particulate debris present in the sample. The supernatant was treated with 1 M NaCl at 4 °C for at least 1 h to release any bacterium-bound phages. The pH of the whey supernatant was adjusted to **~**4.6±0.05 using 0.1 M HCl or 0.1 M NaOH. The whey supernatant was centrifuged at 28,000 ***g*** for 15 min (4 °C). The resulting supernatant was filtered using a double filtration step: first with a 0.45 µm filter, followed by a 0.2 µM filter (Sarstedt). The viral component of the whey filtrate was precipitated with 10% (w/v) PEG 6000 overnight at 4 °C. Precipitated virions were collected by centrifugation at 15,000 ***g*** for 15 min (4 °C), and the pellet was resuspended in 1 ml saline magnesium (SM) buffer (50 mM Tris-HCl, 100 mM NaCl, 10 mM MgSO_4_ and 10 mM CaCl_2_ [23]). This suspension was treated with DNase I (20 U ml^−1^; Millipore Sigma Aldrich) for 15 min at room temperature, to remove any contaminating DNA, followed by DNase inactivation at 75 °C for 10 min. Viral DNA isolation was then performed using the Norgen Biotek Phage DNA isolation kit (Norgen Biotek, Thorold, ON, Canada) according to the manufacturer’s instructions. Viral DNA was quantified using the Qubit dsDNA HS Assay Kit with Qubit 2.0 Fluorometer (Invitrogen, Waltham, MA, USA) according to the manufacturer’s guidelines.

Sequencing libraries were prepared using the Nextera XT DNA Library Preparation kit (Illumina, CA, USA), following the manufacturer’s guidelines. One nanogramme of DNA was used for library preparation undergoing fragmentation and amplification. Paired-end (300 cycles, 2×150 bp) sequencing was performed by GenProbio srl (Parma, Italy) on a NextSeq platform (Illumina). Phageomes were assembled employing two different assembly and annotation methods. For the first method, viral DNA was quality filtered to remove low-quality reads and improve the final quality of the sequenced paired-end reads prior to genome assembly. Using these filtered FASTQ files, phage contigs were assembled and annotated using the METAnnotatorX2 pipeline [39]. The second method employed a bioinformatic tool, Phables, which resolves phage genomes from viral metagenomic assemblies [22]. Prior to running Phables, an assembly graph was generated from the FASTQ files using the open-source assembler SPAdes v3.15.4 [22]. The reads and subsequent SPAdes-generated assembly graph (in Graphical Fragment Assembly format) were submitted to the Phables pipeline (https://github.com/Vini2/phables) to generate the most probable combinations of varying phage genome segments (using read mapping information, graph algorithms and flow decomposition techniques) using default parameters [22]. From the ‘all_phage_like_edges.fasta’ output assembly file (containing sequences from all phage-like components, both resolved and unresolved), ORFs were predicted using Prodigal v2.6.3 prediction software [40]. ORFs were annotated using blastp 2.6.0+ [41] against the non-redundant protein databases curated by the NCBI (https://ncbi.nlm.nih.gov).

The raw virome reads were deposited in the Sequence Read Archive (SRA) under BioProject no. PRJNA1176342, and individual BioSample accession numbers for each virome are listed in Table 1.

### Bioinformatic analysis of RBP sequences

*Skunavirus* RBP sequences were identified from the phageome assemblies based on blastp analysis (Table S1). RBP head domain protein homology detection and initial structure prediction were determined using HHpred (Homology detection and structure production by HMM-HMM comparison) [42] using default settings. Sequence alignments of RBP head domains (C-terminal ~110 aa residues of RBP) were performed using MultAlin [43], employing phageome-identified *Skunavirus* RBP sequences and previously sequenced *Skunavirus* RBPs retrieved from the NCBI virus sequence database (https://www.ncbi.nlm.nih.gov/labs/virus/). Sequence comparison of RBP head domains was performed by all-against-all, bi-directional blast alignment with an alignment (*E*-value) cut-off value of <0.0001 and greater than 50% identity across at least 50% of the aa sequence. ClustalW multiple alignments were conducted to compare aa sequences of *Skunavirus* RBP head domains, and a phylogenetic tree of sequences was constructed using ClustalW (bootstrapped with 1,000 replicates) and visualized via Interactive Tree of Life (iTOL) [44].

### Structural predictions of RBPs using AlphaFold

Structural predictions were performed with either a Colab notebook running AlphaFold v2.3 [36,37], HPC resources from GENCI-IDRIS running AlphaFold v2.3.1 [45] or by AlphaFold3 on Google servers (https://golgi.sandbox.google.com) [46]. The predicted local distance difference test (pLDDT) values (Fig. S1) of predicted structures were stored in the protein database (PDB) files as B-factors and plotted with the Colab or the IDRIS resources. The final predicted protein or domain structures were submitted to the Dali server [45,47] to identify the closest structural homologues in the PDB. Visual representations of the structures were prepared with ChimeraX [48].

### Phageome analysis

To determine the relative abundance of phages present in the assessed whey samples, a phage database was established using publicly available and taxonomically classified lactococcal and dairy streptococcal phage genomes retrieved from NCBI viruses (https://www.ncbi.nlm.nih.gov/labs/virus/). The phage database encompassed a total of 1,155 (640) lactococcal and (515) dairy streptococcal phage sequences, sorted according to their genera (10 lactococcal and 5 dairy streptococcal genera). This database was used to quantify the number of reads (from the raw, FASTQ files quality filtered using NGS QC Toolkit v2.3.3 [40]) that align against each phage genus. CoverM v0.4.0 (https://github.com/wwood/CoverM) was used to determine the proportion of reads that align against each of the mapped phage groups. The relative abundance of phages was determined using the following arguments: coverm genome --methods relative_abundance --min-read-percent-identity 80 --min-read-aligned-percent 50. The relative abundance of *Skunavirus* RBP genes present in each sample was determined through read mapping of a representative RBP-encoding nt sequence of each *Skunavirus* RBP group/subgroup (refined and expanded *Skunavirus* RBP groups will be described below) CoverM v0.4.0 by calculating the reads per kb of exon per million reads mapped using the following arguments: coverm contig --methods rpkm --min-read-percent-identity 86 --min-read-aligned-percent 95 --min-covered-fraction 55.

### Molecular cloning

Primers used in this study (Table S2) were synthesized by Eurofins Genomics (Ebersberg, Germany). PCR amplifications were performed using Phusion High Fidelity DNA polymerase (Thermo Fisher Scientific, Waltham, MA, USA). Primer pairs were used to amplify RBP-encoding genes (list of oligonucleotides and targeted ORFs are presented in Table S2) using whey-extracted virome DNA as a template. PCR products were purified using GenElute^™^ PCR Clean-Up Kit (Millipore Sigma Aldrich). Purified PCR products were cloned in the GFP fusion vector pHTP9 using the NZYEasy Cloning Kit IX (NZYTech genes and enzymes, Lisbon, Portugal) according to the manufacturer’s instructions and introduced into competent *Escherichia coli* BL21 (DE3) by heat shock at 42 °C for 40 s and plated on Luria-Bertani (LB) agar (BD Difco, Oxford, UK) supplemented with 50 µg ml^−1^ kanamycin (Millipore Sigma Aldrich) [49]. The integrity of recombinant plasmid sequences was verified using Sanger sequencing (GeneWiz, Leipzig, Germany) using vector (pHTP9-F GAATGAAAAACGCGACCACATGGTG and pHTP-R GGTTATGCTAGTTATTGCTCAGCG) and/or insert-specific primers.

### Recombinant protein production

Aliquots (2 ml) of *E. coli* BL21 (DE3) carrying recombinant plasmids of interest were inoculated in 200 ml LB medium supplemented with 50 µg ml^−1^ of kanamycin and 10% glycerol followed by incubation at 37 °C in a shaking incubator (Infors HT Multitron Standard, Bottmingen, Switzerland) at 150 r.p.m. until cultures reached an OD_600 nm_ between ~0.3 and 0.7. Proteins were then induced with 0.05 mM IPTG (Thermo Fisher Scientific) and incubated overnight at 20 °C in a shaking incubator (Infors HT Multitron Standard) at 250 r.p.m. Cells were harvested by centrifugation at 4,600 ***g*** for 30 min and resuspended in 10–20 ml (depending on the size of cell pellets) of lysis buffer (50 mM Tris-HCl, 300 mM NaCl, 10% glycerol, 1% Triton X-100 and pH 7.5) and incubated shaking at 4 °C for at least an hour. The cells were disrupted by sonication in an MSE Soniprep (Sanyo, Osaka, Japan) at maximum amplitude for five 30-s cycles followed by a 30-s rest, with a holding step on ice for at least 2 min following the third round of sonication. Proteins were separated from cell debris by centrifugation at 15,000 ***g*** for 30 min (4 °C). His-tagged proteins were column purified with standard Ni-NTA agarose (Qiagen, Manchester, UK) in protein buffer (50 mM Tris-HCl, 300 mM NaCl, 50 mM CaCl_2_ and pH 7.5) with increasing concentrations of imidazole (50–250 mM). The size and purity of the GFP-RBP fusion proteins were assessed by SDS-PAGE. Protein concentrations were quantified using the Qubit Protein Assay Kit with Qubit 2.0 Fluorometer (Invitrogen) according to the manufacturer’s guidelines. Purified proteins were dialysed against protein buffer stored at 4 °C.

### GFP-RBP-host binding assays and fluorescence microscopy

Binding activity assessment of the His-tagged GFP-RBP fusion proteins (^His^GFP-RBP) was performed with cells of various lactococcal strains (Table 2), which had been grown to mid-log phase (OD_600 nm_ ⁓0.4–0.7), harvested by centrifugation at 10,000 ***g*** for 5 min and resuspended in an equal volume of SM buffer. One hundred microlitres of the bacterial resuspension was mixed with 12.5 µg of each GFP-RBP fusion protein in protein storage buffer, added to a well in a 96-well round (U) bottom plates (Thermo Fisher Scientific) containing 100 µl of SM buffer and incubated at 30 °C for 12.5 min. Control wells containing only 12.5 µg of ^His^GFP-RBP in SM buffer were included in each assay. Cells were centrifuged (2,000 ***g*** for 5 min) and washed three times with 200 µl of SM buffer. Prior to fluorescence quantification, the OD_600 nm_ was measured with a microplate reader (Multiskan™ FC Microplate Photometer, Vantaa, Finland). Subsequently, the samples were transferred to black polystyrene 96-well plates (Thermo Fisher Scientific) where fluorescence was quantified with a Tecan Infinite 200 PRO series plate reader (Tecan Group Ltd., Männedorf, Switzerland). GFP-RBP fusion proteins were excited at a wavelength of 488 nm, with a z-stack of 20,000 µm and a gain of 80. Arbitrary fluorescence units (FUs) were determined with the following formula: $\frac{SampleFU-controlwellFU}{OD600nmofsample}$. A relative binding affinity score was then calculated as follows: AverageFUofaGFP∷RBPHistoastrainAverageFUofaGFP∷RBPHistoaoptimalhoststrain×100%, where the optimal host strain is the strain to which a given ^His^GFP-RBP consistently binds with the highest affinity.

For fluorescence microscopy, 6 µl of lactococcal cell suspensions labelled with fluorescent ^His^GFP-RBP (as described above) was transferred onto a glass slide and covered with a cover slip. Fluorescence imaging was performed with an Olympus BX53 fluorescence microscope (Olympus, Tokyo, Japan) using CellSens software (Olympus), a 60 or 100× objective lens and a U-FBNA filter (barrier filter: 470–495 nm, dichroic mirror: 505 nm).

### Isolating novel phage–host combinations through PhRACS

Sorting of ^His^GFP-RBP_AO-4_ and ^His^GFP-RBP_BQ-1_ was performed in the same manner as described above for the binding assays, using activated bulk starter culture M1 (using the so-called PhRACS method as previously described [23]). M1 is an undefined mesophilic industrial starter culture commonly used in the production of semi-hard cheeses, fermented milk and fresh cheeses. Ten grammes of lyophilized M1 were diluted in 207 g of sterilized (115 °C for 20 min) 10% (wt/vol) reconstituted skimmed milk powder. Following homogenization in a Stomacher 400 Circulator (Seward Ltd., Worthing, UK) for 2 min at 230 r.p.m., this mixture was inoculated (at 2%) into LM17 (Millipore Sigma Aldrich) and incubated overnight at 22 °C. LM17 was inoculated overnight and incubated at 30 °C until it reached an OD_600 nm_≈0.3–0.4. ^His^GFP-RBP fusion protein-labelled bacteria were sorted with a BD FACSARIA Fusion cell sorter with 85 µM nozzle and a 488 nm laser with 530/30 filter for GFP excitation and detection. GFP-positive events were sorted as single cells into 96-well plates containing 150 µl of LM17. Ninety-six-well plates were then incubated at 30 °C for 48 h. Isolates were subcultured into 96-well plates containing LM17, grown overnight and stored at −20 °C in 25% glycerol. The *cwps* genotype of recovered isolates was determined using previously defined *cwps*-type specific multiplex PCR systems [26,27].

## Results

### Phageome of dairy-derived whey samples

**Fig. 1.**
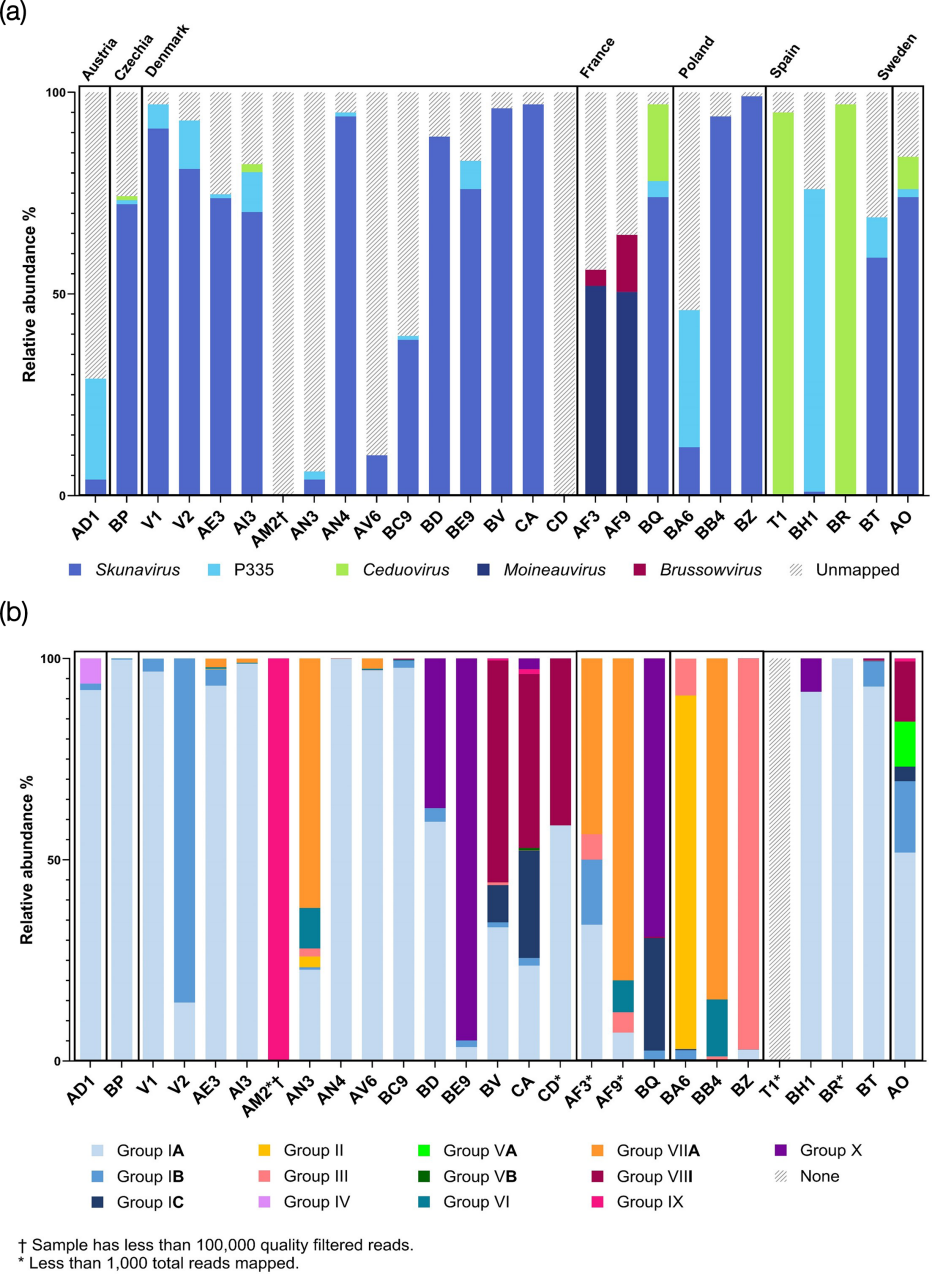
Phage diversity present in whey virome samples from dairy fermentation factories across Europe based on read mapping. (**a**) Relative abundance of lactococcal (*Skunavirus*, P335 and *Ceduovirus*) and streptococcal phages (*Moineauvirus* and *Brussowvirus*). (**b**) Relative abundance of *Skunavirus* RBP groups. Reads that do not map to any of the phage database or RBP groups, either due to novel phage sequences (not present in any reference genome) or contaminating DNA (mainly bacterial), are depicted as diagonally striped grey lines. Further details regarding the relative abundance and total number of reads (in a given sample) that mapped against RBP groups can be found in Table S5.

To gain deeper insights into phage–host interactions occurring in dairy fermentation factories employing mesophilic undefined starter cultures, the viral fraction of 27 whey samples collected from several factories across Europe utilizing mesophilic undefined starter cultures (typically supplemented with additional strains/cultures) was extracted and sequenced. An average sequencing output of 3,666,334 high-quality reads per sample was generated (Table S3). To assess the phage prevalence and diversity of these whey samples, the sequencing reads were mapped against an in-house database of lactococcal and *Streptococcus thermophilus* reference phage sequences (Fig. 1a). This mapping demonstrated that skunaviruses are the dominant phage group in 18 of the 27 analysed whey samples, followed by the P335 (3 samples) and ceduoviruses (2 samples). In two whey samples collected in France, AF3 and AF9, streptococcal phages belonging to the *Moineauvirus* and *Brussowvirus* genera were the most dominant. The presence of streptococcal phage sequence in these samples may emanate from phages associated with fermentations incorporating *S. thermophilus-*containing starter cultures within the factory (i.e. cross-contamination from parallel thermophilic fermentation) or may indicate that *S. thermophilus* strains were added to supplement or support the mesophilic fermentation. The percentage of reads that did not align to any of the reference genomes varied greatly (1–100%), mainly due to contaminating bacterial DNA or (in fewer cases) the presence of non-lactococcal/streptococcal phage genomes (Table S4). Reads were assembled using the METAnnotatorX2 and Phables assembly pipelines, and select *Skunavirus* contigs (confirmed through blastn analysis) were investigated for further analysis as described below.

### Analysis of *Skunavirus* RBPs

**Fig. 2.**
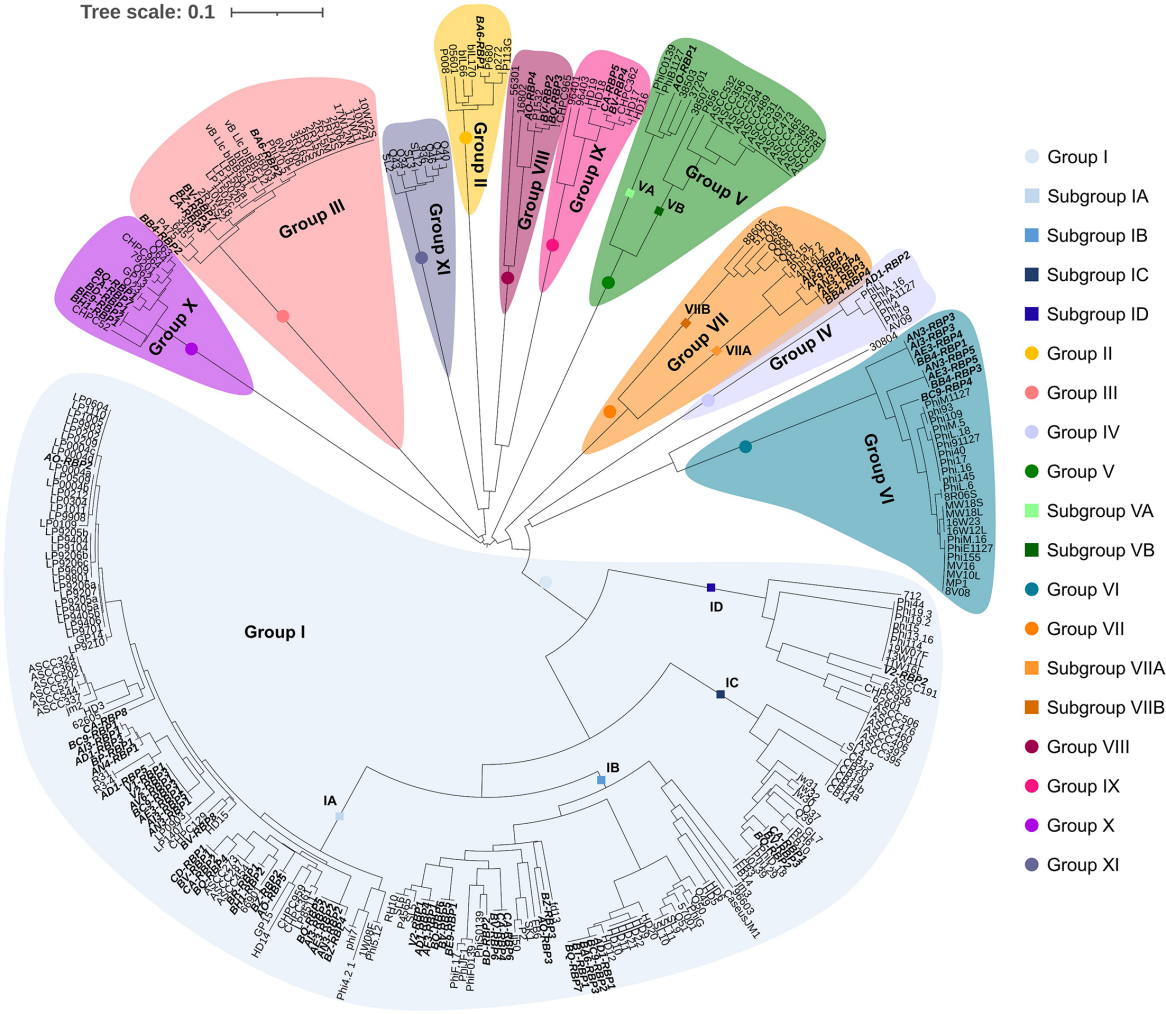
Phylogenetic tree of the C-terminal region or head domain of identified RBPs from whey phageome-derived assemblies (written in bold) alongside publicly available *Skunavirus* RBP sequences. aa sequences of *Skunavirus* RBP head domains identified from whey phageomes (81 RBP sequences) together with those encoded within publicly available *Skunavirus* genomes, 332 RBPs in total, were aligned and grouped together based on sequence similarity.

Since members of the *Skunavirus* genus were shown to greatly dominate the phageome in the majority of examined whey samples, it was decided to exclusively focus on these phages. As RBP-mediated adsorption of a *Skunavirus* to its host is the first step in the infection process, the obtained phageome sequences were scrutinized for predicted *Skunavirus* RBP-encoding genes. Using METAnnotatorX2- and Phables-assembled *Skunavirus* contigs, 81 *Skunavirus* RBP sequences (after dereplication within a given sample) were identified using blastp. *Skunavirus* RBP sequences are known to exhibit considerable similarity within the first ~135 N-terminal aa residues, which correspond to the shoulder and neck domains of the RBP, while considerable variation is observed in the remaining ~110 C-terminal residues, representing the RBP head domain, which interacts with the host receptor represented by the CWPS. A previous study of 91 *Skunavirus* RBP sequences had identified five RBP groups (I–V) based on varying similarity levels that were found to correspond to phage host CWPS preference [26,28]. With access to 332 RBP sequences (251 publicly available RBPs and the 81 phageome-derived RBPs generated as part of this study), a refinement and expansion of the previously defined *Skunavirus* RBP groups was established through comparative analysis of the C-terminal region (~110 residues of the C-terminal end of each RBP) of all (i.e. 332) currently available *Skunavirus* RBPs. Multiple aa sequence alignments of the individual RBP head domains of these 332 RBPs were performed, as outlined in the ‘Methods’ section (Figs S2 and S3), and a phylogenetic tree was generated to visualize varying RBP groups (Fig. 2). Through this analysis, 11 distinct RBP groups were identified (as well as the presence of several subgroups). RBP groups were strictly defined here based on a particular group sharing a minimum of 50% aa identity across 90% of the RBP head domain with other group members. aa identity within the head domain of members of a particular group was typically considerably higher than the minimum threshold (between ~82 and 95% identity). However, for groups where there was an observed divergence in the aa sequence of the head domain among group members (but still sufficient identity to fall within a single group based on the defined parameters), subgroups were defined in which RBPs shared at least 65% of aa residues within the head domain of all subgroup members (a cut-off that was subsequently shown to correspond to differences in host binding and/or AlphaFold3-mediated structural predictions, see below). Our RBP head analysis revealed the presence of 11 distinct RBP groups (designated here as groups I through to XI) and with subgroup recognition in the case of groups I (four subgroups, designated IA through to ID), V (two subgroups: VA and VB) and VII (two subgroups: VIIA and VIIB), representing a significant expansion of the previously defined five RBP phylogenetic groups. One *Skunavirus* member, i.e. phage 30804, possesses an RBP, which does not fall within any of the groups based on the phylogenetic analysis and may thus represent an additional group to be expanded in future studies. The relationship between the proposed expansion and associated refinement of RBP groups is presented in Table 3 and will be elaborated upon below. Four of the here identified 11 RBP groups (i.e. groups I–III and V) correspond to one of the previous groups (albeit with the recognition of subgroups for groups I and V), whereas the previous group IV has now been redefined to represent two groups (groups IV and VI), based on the defined parameters, while the remaining five groups are novel (Table 3).

**Table 3.** RBP groups as determined through phylogenetic analysis. Correlation to the previously described RBP grouping system and preference of an RBP group to a particular CWPS(s) is described

Group/subgroup	Relation to previous RBP grouping*	CWPS specificity†
Subgroup IA	Group I	C (C₂, C₁)‡
Subgroup IB		C*
Subgroup IC		C (C₄, C₅)‡
Subgroup ID		C (C₉, C₂, C₁)‡
Group II	Group II	B
Group III	Group III	B and C*
Group IV	Group IV	U
Subgroup VA	Group V	A*
Subgroup VB		
Group VI	Group IV	D
Subgroup VIIA	Novel	U
Subgroup VIIB	Novel	U
Group VIII	Novel	C₆
Group IX	Novel	U
Group X	Novel	C₃
Group XI	Novel	U

*Host specificity determined in previous studies [26,28].

†Unknown CWPS specificity is indicated by ‘U’.

‡Preference for particular C subtypes determined through fluorescent binding assays, affinity to a particular subtype listed in descending order.

The 81 phageome-derived RBPs identified in the current study were distributed among 10 of the 11 here identified RBP groups, as presented in Table S1, where individual RBPs are named according to the whey from which they originated and numbered sequentially. The majority (50 of 81) of phageome-identified RBP sequences are members of RBP group I, thus representing the most prevalent RBP group in the assessed phageomes (Fig. 2). All group I RBPs share the aa Trp 144, Arg 256, Asp 234 and His 232 (Fig. S2) from the RBP head of phage p2, residues which have previously been determined to be located within its sugar-binding crevice [31,32, 34]. A previous study had suggested that additional aa residues (other than the four conserved residues mentioned above) impact the specificity of RBP binding, justifying the decision to introduce subgroups (being supported by differences in host specificity of subgroups, which will be discussed below) [50]. The remaining phageome-derived RBPs belong to (sub)groups II, IV or VA (one RBP identified for each of these); group IX (two); group VIII (three); and (sub)groups III, VIIB and X (five each), and eight were found to belong to group VI. Read mapping was performed to determine the relative abundance of each of the redefined *Skunavirus* RBP (sub)groups (Fig. 1b and Table S5). Of note, group I RBPs (specifically subgroup IA) were found to be the most prevalent in 14 of the 27 analysed whey samples and present at some abundance (>2%) in all but 5 samples (AM2, T1, BQ, BA6 and BB4; Fig. 1b). A notable trend among the Danish whey samples is that novel RBP groups VIII and X began appearing in samples collected in 2021 and progressively became more dominant in samples collected in 2022 and 2023. In this context, it should be noted that group I RBPs were the most dominant in most (7/11) Danish samples collected between 2019 and 2021 (apart from AM2, AN3 and BE9). Of further note, lactococcal RBPs (belonging to groups I, VI or VII) are present in two whey samples (AF3 and AF9) collected in France (Fig. 1b and Table S1) despite streptococcal phages being the dominant phage genera in these samples (Fig. 1a).

### Structural predictions of novel RBPs

**Fig. 3.**
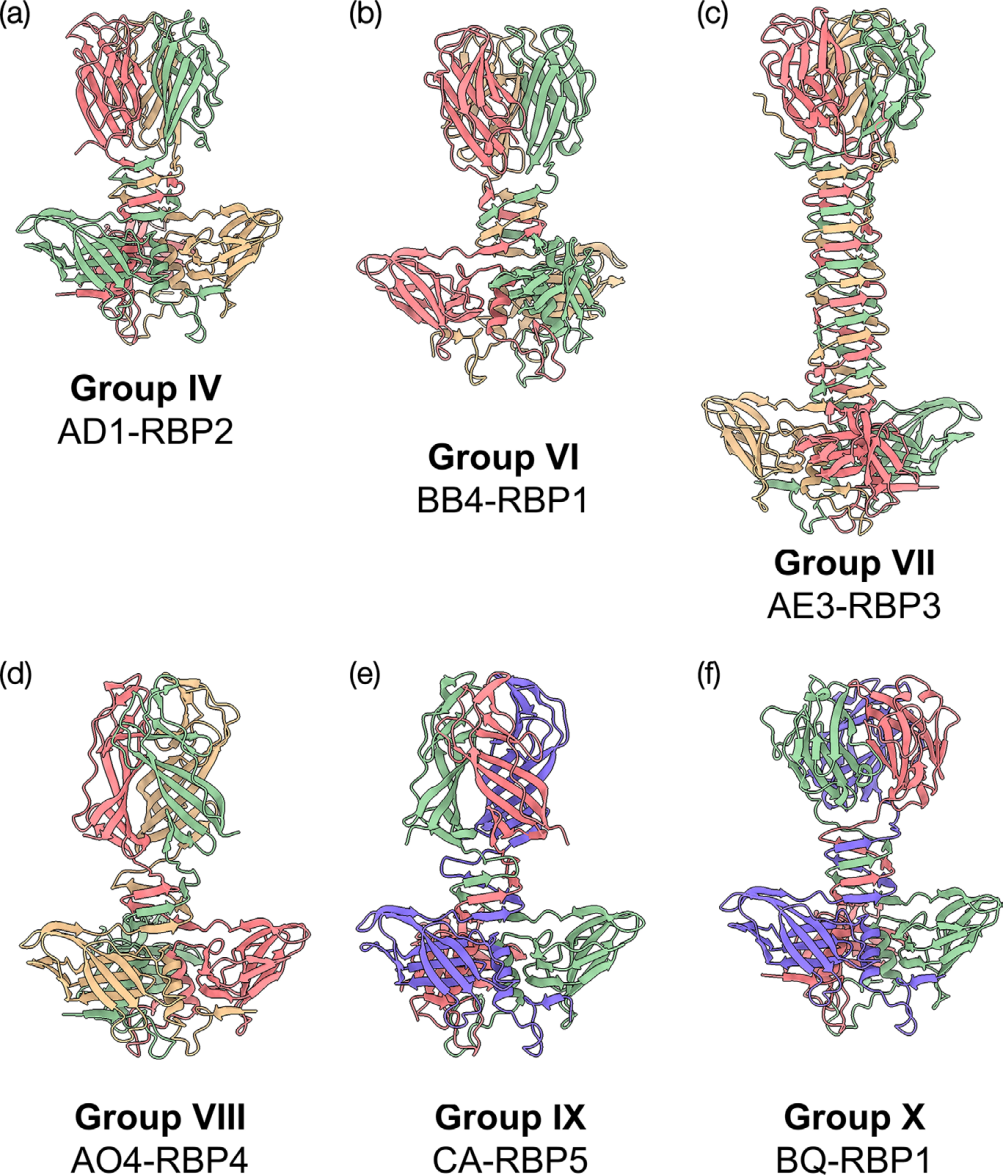
Structural analysis of selected members of newly recognized RBP groups (IV and VI–X). Structures of representatives of the novel and redefined group IV as determined with AlphaFold2: (**a**) AD1-RBP2 (group IV), (**b**) BB4-RBP1 (group VI), (**c**) AE3-RBP3 (group VII), (**d**) AO-RBP4 (group VIII), (**e**) CA-RBP5 (group IX) and (**f**) BQ-RBP1 (group X).

To determine the impact of the sequence diversity exhibited by the phageome-derived RBPs on the structural complexity and diversity of *Skunavirus* RBPs, six phageome-derived RBPs were selected as representatives of their groups (IV and VI–X) for structural analysis using AlphaFold2 (Fig. 3), expanding the previous structural analysis of members of the other four RBP head domain groups (I–III and V) [31]. All six RBP group representatives possess the conserved modular RBP structure observed in all skunaviruses, consisting of the head, neck and shoulder domains. Furthermore, group VII (subgroup VIIB) representative AE3-RBP3 exhibits a distinct structure including a very long neck domain (Fig. 3c), similar to those of lactococcal P335 phages, LC3 and Q33, although not every member of this group possesses a similarly extended neck domain. Of the six novel RBP structures, only the representative RBP head domain of group IV (AD1-RBP2) shares residues (Asn 234 and Arg 255/256) located in the binding crevice of the RBPs of phages p2 [32,34] and TP901-1 [51] (Fig. S4). The head domains of the selected members of groups IV, VI and VII and groups VIII and IX, respectively, appear to share similar folding characteristics to each other (Fig. 3d, e), yet have different aa residues in the binding crevice (Fig. S3). Additionally, the group X representative RBP sequence, BQ-RBP1, exhibits an apparently unique folding pattern within its head domain when compared to the other RBPs (Fig. 3f).

### Fluorescent binding assays employing His-tagged GFP-RBP fusion

**Fig. 4.**
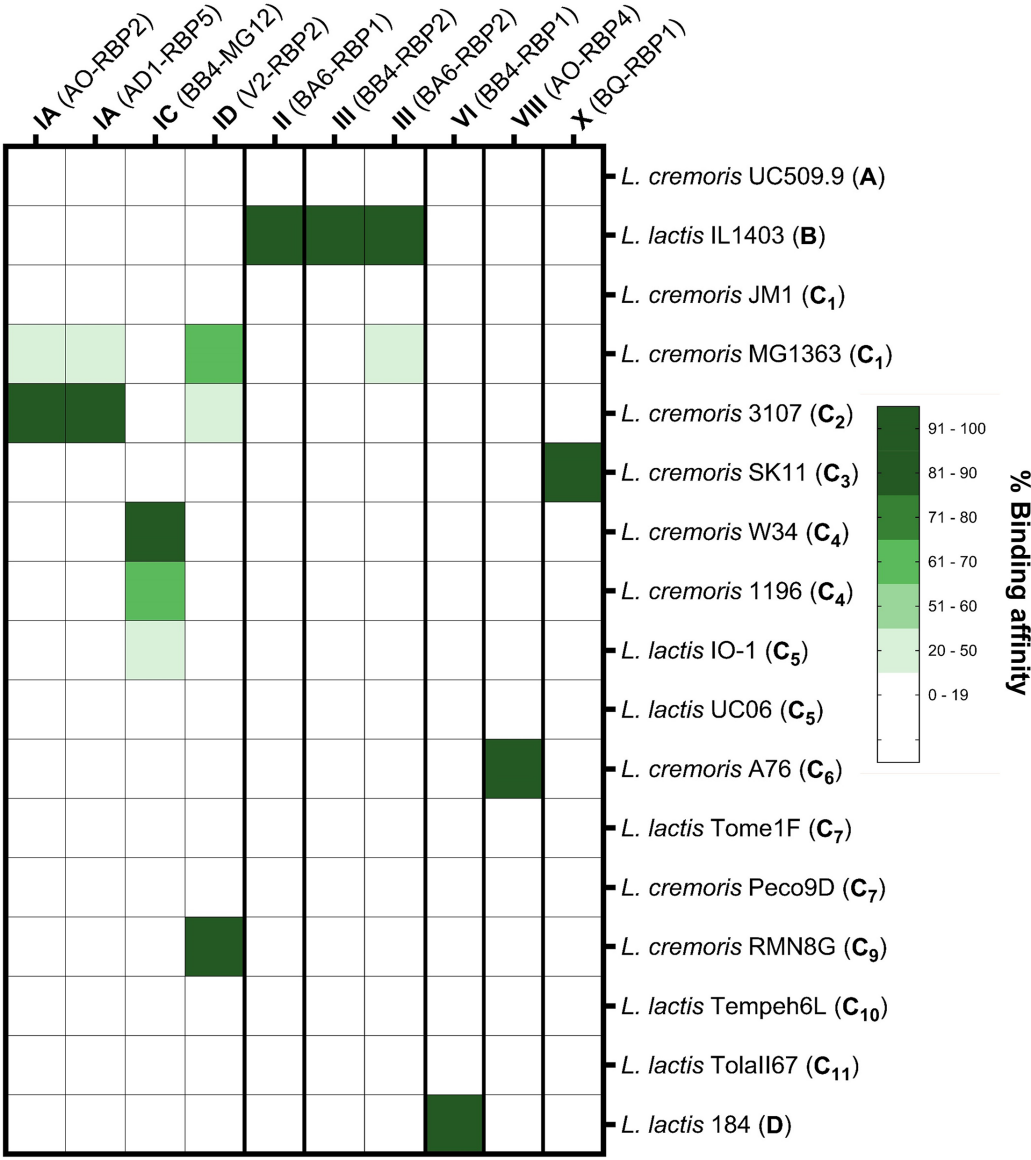
Binding abilities of *Skunavirus* RBP-GFP fusions to lactococcal strains of various CWPS types. A relative binding affinity score for each ^His^GFP-RBP to lactococcal strains representing the various *cwps* genotypes was calculated, according to the ‘Methods’ section (i.e. as a ratio of the average fluorescence unit against a particular strain to that of the optimal host strain corresponding with the highest affinity). Detailed information regarding the fluorescence binding assay of the ^His^GFP-RBPs is presented in Fig. S5. Additional information regarding both the symbolic and full chemical representations of the surface-exposed side chain component of lactococcal strain(s) (as determined by previous studies [25,27, 30, 68]) to which a particular ^His^GFP-RBP was able to bind can be found in Fig. S8.

To experimentally explore the specificity of RBP-mediated binding to lactococcal CWPS among the phageome-identified RBPs, we selected 15 distinct RBP-encoding genes, representing RBP groups I–X (and more specifically subgroups IA, IC, ID, VA and VIIB), cloned these into the expression vector pHTP9 and created constructs for the production of GFP-RBP fusion proteins of ~54–64 kDa (see the ‘Methods’ section; Table S2). In all cases but one (RBP_BB4-MG12_, originating from a *Skunavirus* genome from an in-house phage collection), RBP sequences were selected from phageome sequence assemblies. These RBP-GFP fusion proteins were used in binding assays employing available lactococcal strains representative of 13 currently defined *cwps* gene clusters (CWPS types A, B, C_1_-C_7_, C_9_-C_11_ and D). Overall, it was possible to determine the binding specificity of 10 of the 15 GFP-RBP fusion proteins [representing eight RBP (sub)groups]; the results obtained from these binding experiments are summarized in Fig. 4 and will be discussed in further detail below.

### Binding specificity of group I, II and III RBP representatives

**Fig. 5.**
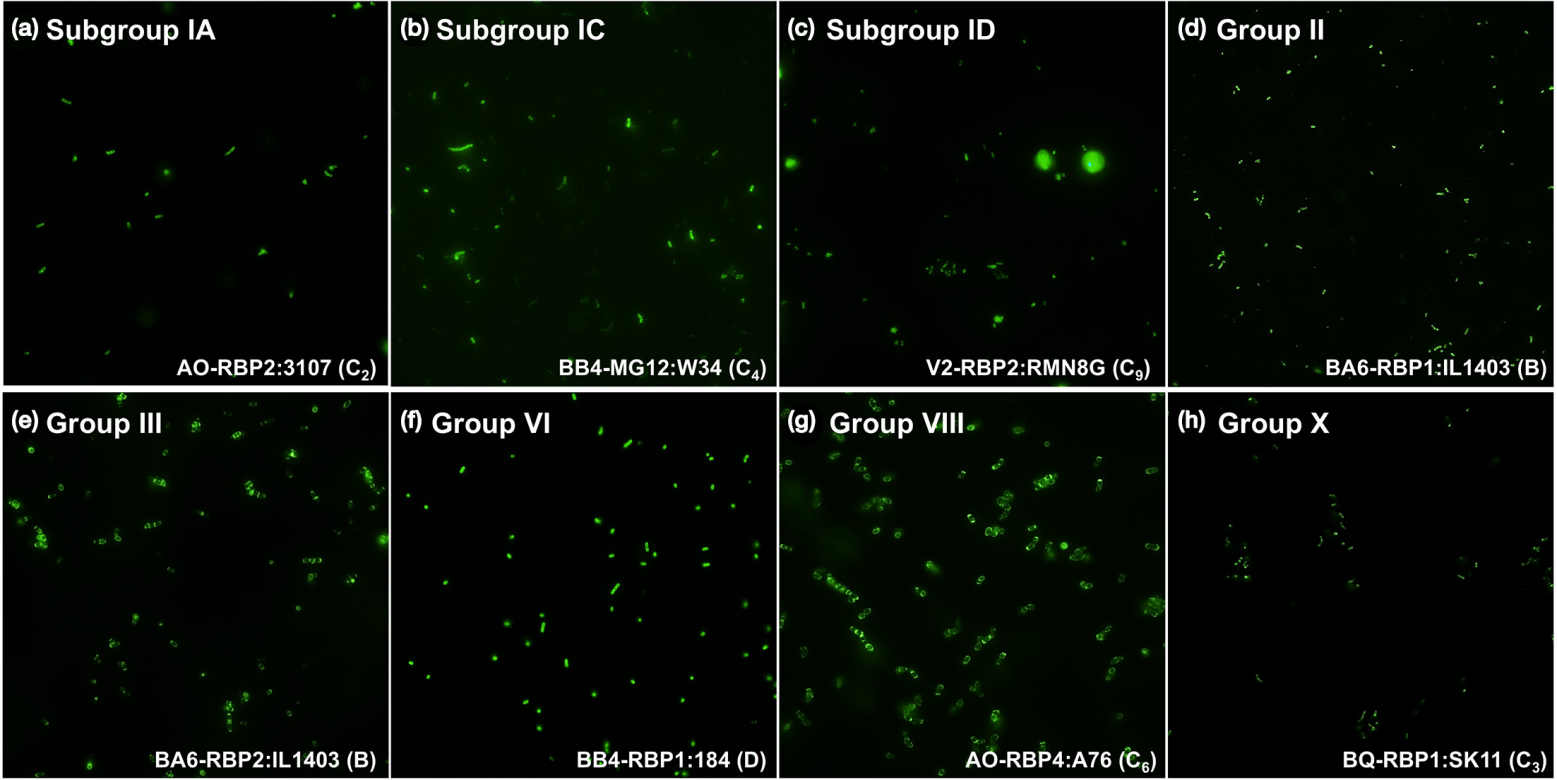
Fluorescence imaging of ^His^GFP-RBPs representing eight (sub)groups to various lactococcal CWPS-type strains. (**a–h**) Fluorescence imaging of ^His^GFP-RBPs representing groups I (subgroups IA, IC and ID) II, III, VI, VIII and X to indicate lactococcal strains (with highest binding affinity).

**Fig. 6.**
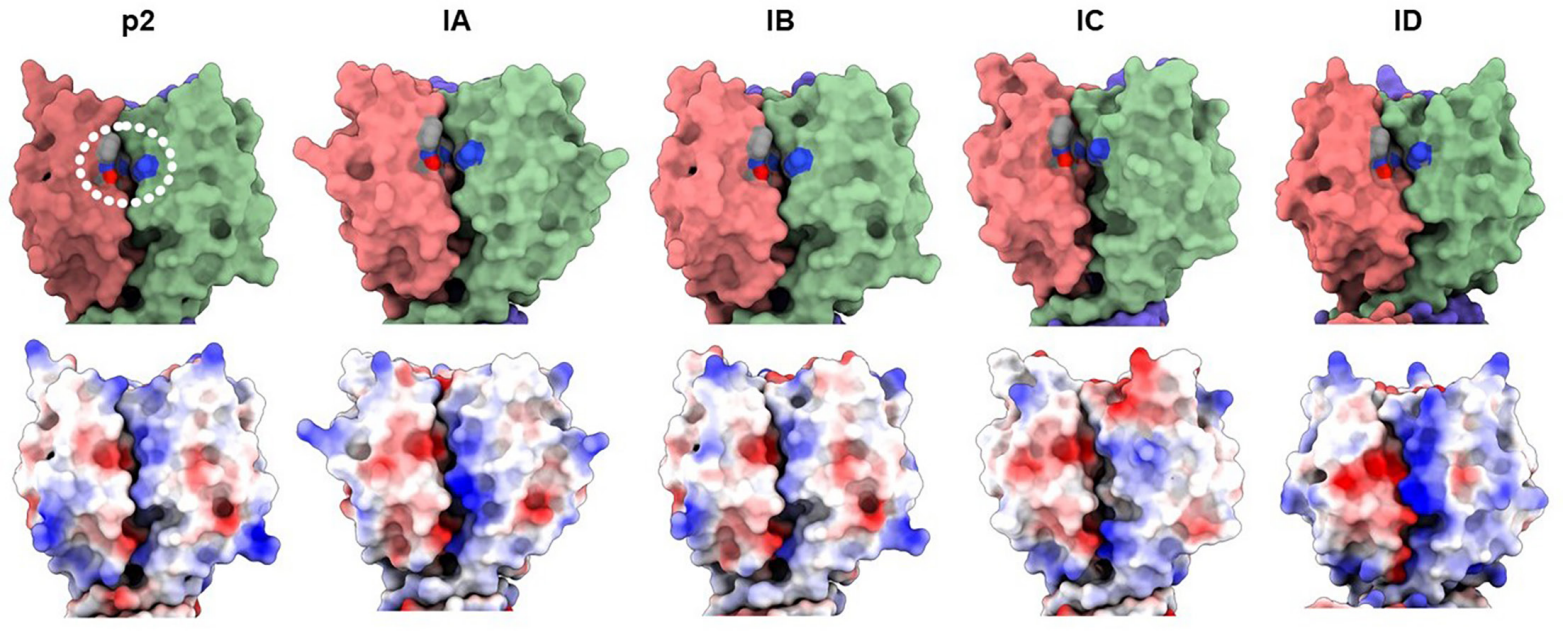
Structure predictions of group I RBPs. Structures of representatives of the subgroups IA to ID as determined by AlphaFold3 compared to phage p2 head domain. In the primary binding site of the RBP head domain (circled in the p2 head domain), all group I RBPs share four conserved aa residues (Trp 144, Arg 256, Asp 234 and His 232) with *Skunavirus* p2. There are differences in terms of both shape and electrostatic potential between the group I RBP subgroup representatives. The electrostatic potential is represented by different colours: blue, positive; red, negative; and white, neutral.

Binding assays were performed using 17 lactococcal strains representing *cwps* types A, B, C_1_-C_7_, C_9_-C_11_ and D. Of the 17 strains, subgroup IA representatives, ^His^GFP-RBP_AO-2_ and ^His^GFP-RBP_AD1-5_, were both shown to be capable of binding to strain 3107 (C_2_) with high affinity, at 1.50×10^5^ and 1.81×10^5^ FU, respectively, and to strain MG1363 (C_1_) though with significantly lower affinity, at 5.57×10^4^ and 4.10×10^4^ FU (*P*-value<0.01; Figs 5a and S5 A&B). Subgroup IC representative, ^His^GFP-RBP_BB4-MG12_, was shown to predominantly bind to C_4_-type strain W34 at 2.08×10^5^ FU and with decreased binding to C_4_-type strain 1196 at 1.29×10^5^ FU and C_5_-type strain IO-1 at 6.88×10^4^ FU (Figs 5b and S5 C). ^His^GFP-RBP_V2-2_ (subgroup ID) is capable of binding to both MG1363 (C_1_) at 6.50×10^4^ FU and 3107 at 4.13×10^4^ FU, while it binds to strain RMN8G (C_9_) with significantly higher affinity at 1.07×10^5^ FU (*P*-value<0.0001; Figs 5c and S5 D). Structure predictions of the subgroup I RBP heads revealed clear differences between the head domains, both in terms of shape and electrostatic potential, likely explaining the distinct binding characteristics of the different RBP head domains (Fig. 6).

Group II (^His^GFP-RBP_BA6-1_) and group III (^His^GFP-RBP_BB4-2_ and ^His^GFP-RBP_BA6-2_) representative RBPs specifically recognize and bind to the *cwps* B-type strain IL1403 at 1.92×10^5^, 2.24×10^5^ and 3.03×10^5^ FU, respectively (Figs 5d, e and S5 E–G). ^His^GFP-RBP_BA6-2_ of group III (Fig. S5 G) was also found to bind to MG1363 (*cwps* C_1_) at a significantly reduced affinity (7.97×10^4^ FU).

### Investigating binding capabilities of emergent RBP groups

Binding assays were conducted on (representatives of) previously unstudied/unknown RBP groups to evaluate the link to their host CWPS type preference of identified potential host strains. Group VI representative ^His^GFP-RBP_BB4-1_ binds specifically to D-type strain 184, at 1.85×10^5^ FU (Figs 5f and S5 H). Group VIII representative ^His^GFP-RBP_AO-4_ binds specifically to C_6_-subtype strain A76 at 5.34×10^4^ FU (Figs 5g and S5 I). Group X representative, ^His^GFP-RBP_BQ-1_, binds to SK11 (C_3_) with high affinity at 1.59×10^5^ FU (Figs 5h and S5 J). Group IV representative RBP (^His^GFP-RBP_AD1-2_), group VII (subgroup VIIA) RBPs (^His^GFP-RBP_AE3-3_ and ^His^GFP-RBP_BB4-4_) and group IX RBP (^His^GFP-RBP_CA-5_) did not appear to bind to any of the CWPS-type strains with particularly high affinity (compared to the other RBPs studied and visually confirmed with fluorescence imaging; Fig. S6).

### Phage–host retrieval using PhRACS

To complement the results of the binding assays, attempts were made to isolate individual phage–host combinations corresponding to the following eight RBPs representing groups II and IV through X (predominantly representing the novel or refined RBP groups) through the PhRACS method: AD1-RBP2, BB4-RBP1, BA6-RBP1, AO-RBP1, AE3-RBP3, AO-RBP4, BQ-RBP1 and CA-RBP5. To isolate novel phage–host combinations from dairy-derived whey samples, ^His^GFP-RBP corresponding to the above RBP-encoding genes identified from phageome data representing several RBP groups were used to identify GFP-positive cells from an undefined mesophilic starter culture (M1) employed in the dairy fermentation systems. Of the RBPs attempted, only with ^His^GFP-RBP_AO-4_ and ^His^GFP-RBP_BQ-1_ (determined to bind specifically to C_6_ and C_3_-type strains, respectively, see previous paragraph) was it possible to successfully retrieve potential hosts through PhRACS. Of the total population of cells, 1.7% and 0.7% of cells were found to be GFP labelled by ^His^GFP-RBP_AO-4_ and ^His^GFP-RBP_BQ-1_, respectively (Fig. S7). From ^His^GFP-RBP_AO-4_ and ^His^GFP-RBP_BQ-1_ sorting, 52 and 83 culturable isolates were successfully retrieved following 24–72 h of incubation at 30 °C, respectively. Forty-two of the 52 ^His^GFP-RBP_AO-4_ sorted isolates were typed as *cwps* subtype C_6_, and the remaining 10 were C_1_-subtype strains. Of the 83 ^His^GFP-RBP_BQ-1_ sorted isolates, 75 were typed as *cwps* subtype C_3_, and the remaining 8 were C_1_-subtype strains. Representative isolates of these subtypes were tested for binding against ^His^GFP-RBP_AO-4_ and ^His^GFP-RBP_BQ-1_ to identify potential hosts. ^His^GFP-RBP_AO-4_ was shown to bind to C_6_ isolates and ^His^GFP-RBP_BQ-1_ to the C_3_ isolates (neither the C_1_ isolates), and these isolates were used in phage isolation from the original whey samples through spot assays, leading to the isolation of skunaviruses AO-4 and BQ-1. Attempts to use PhRACS to isolate potential hosts for BB4-RBP1, BA6-RBP1, AO-RBP1, AE3-RBP3 and CA-RBP5 were unsuccessful, likely as a result of suitable hosts either occurring in very low abundance (below the sensitivity of this approach) or not being present in the starter culture population used in the study.

## Discussion

In recent years, consumer expectations of consistent and high-quality fermentation products have led to an increase in both metagenome and virome studies of various food production systems and products [52,56]. This has generated a wealth of knowledge on the microbiota and community dynamics occurring in various fermentation systems, facilitating more reliable methods of community monitoring. Studies of viral metagenomics of food systems have revealed an abundance of, as yet, unclassified viral ‘dark matter’ (i.e. not aligning to any reference viral sequences), in food systems [57]. Consequently, more targeted virome extractions and phageome-focused bioinformatic analysis methods have been developed [16,19]. In the current study, the viromes of 27 whey samples were successfully extracted/sequenced, and the subsequent phageomes were analysed using 2 different assembly methods to yield as many phage-like contigs as possible. The majority of whey phageomes (i.e. 18 out of 27 assessed samples) were shown to be dominated by lactococcal phage sequences, with members of the *Skunavirus* genus being the principal phages present in almost all assessed samples (Fig. 1a). This is in line with several other (culture-based) studies investigating the diversity of phages associated with dairy fermentation facilities using mesophilic starter cultures [10,58, 59]. However, both T1 and BR whey samples originate from a Spanish factory and were shown to predominantly contain ceduoviruses. Both skunaviruses and ceduoviruses are strictly lytic and represent the most problematic phages in commercial dairy fermentation facilities.

As adsorption is crucial in the initiation of the *Skunavirus* infection process, lactic acid bacteria (LAB) strains have been found to mutate or alter their host receptors [33,60]. In turn, phages may modify or mutate their RBPs or even acquire novel genes that allow them to overcome the modifications to host receptors [50,61,63]. Such systems of modifying RBPs include diversity-generating retroelements, first characterized in *Bordetella* phage BPP-1 [64] and identified in other lysogenic phages infecting various *Bacteroidota*, *Pseudomonadota*, *Bacillota* and *Actinomycetota* [65,66], as well as homologous recombination events between closely related phages or prophages, which have been observed between LAB phages [63,67]. Another RBP diversification system identified in *Bifidobacterium* strains is the Rin shufflon system that generates RBP variants between a constant N-terminus and variable C-terminal RBP regions utilizing a tyrosine-family recombinase [62]. In *Skunavirus* escape mutants that can overcome phage-resistant derivatives of the lactococcal strain 3107 (with reduced synthesis of the CWPS side chain), it appears that mutation to key aa residues likely involved in saccharide binding is an effective strategy used by skunaviruses to counteract host receptor modifications or acquire novel host recognition abilities [50]. Therefore, the diversity of skunaviruses in these factories was the primary focus of the investigation, particularly the diversity of RBPs, which are responsible for the initial binding and recognition of phages to a cognate host.

Group I RBPs are structurally highly conserved (resemble phage p2 RBP) and share four aa residues (Trp 144, Arg 256, Asp 234 and His 232), which have previously been determined to be found within the binding crevice of phage p2 [31], while it has been suggested recently that additional residues are involved in saccharidic binding [50]. Detailed analysis of the head domain of group I RBPs present in the whey phageomes led to the identification of notable aa differences within the RBP head domains. Consequently, group I RBPs were divided into four subgroups. Representative RBPs from three of the four group I subgroups (i.e. subgroups IA, IC and ID) were used to generate ^His^GFP-RBPs that were subsequently tested for their ability to bind to lactococcal strains representing 15 distinct CWPSs (A, B, C_1_-C_7_, C_9_-C_11_ and D). Here, we confirmed that group I RBPs bind specifically to C-type *cwps* strains and that differences in key aa residues in the sugar-binding domain are linked to a binding preference for certain C-subtype strains. In the case of subgroup IA, this appears to be C-subtype C_2_ (and comparatively low affinity to C_1_); for subgroup IC, it is C-subtype C_4_ (and comparatively low affinity to C_5_); and for subgroup ID, it is C-subtype C_9_ (with low affinity to C_1_ and C_2_-types). This aligns with previous host range studies of a number of skunaviruses (included in the RBP group analysis) possessing group I RBPs that have been found to infect hosts possessing various *cwps* C genotypes [26,28]. The CWPS of previously analysed C subtypes has been found to comprise a linear rhamnan backbone with repeating oligosaccharide or polymeric side chains [25,68]. It is now possible to link specific group I RBP subgroups to the considerable variation in the number and composition of monosaccharides (and chemical modification of these monosaccharides) making up the C subtype side chain(s) capable of binding (as illustrated in Fig. S8).

Both the group II representative ^His^GFP-RBP_BA6-1_ and group III representatives (^His^GFP-RBP_BB4-2_ and ^His^GFP-RBP_BA6-2_) were found to bind to B-type strain IL1403, aligning with previous CWPS preferences of phages within these groups [26,31]. However, certain skunaviruses possessing group III RBPs have also been shown to have the ability to infect C-type lactococcal strains (based on host range studies) [26,31]. The binding affinity of the subgroup VA ^His^GFP-RBP representative could not be confirmed through fluorescent binding assays; however, previous studies have found that skunaviruses possessing group V RBPs are capable of infecting lactococcal strains containing an A-type *cwps* gene cluster [26,31]. As the mesophilic starter cultures used in starter culture rotation systems in these fermentation facilities may be (or are derived from) starter cultures primarily composed of various *cwps*-type C lactococcal strains [56], it is likely that these phages infect strains that have been added to supplement the starter cultures in these facilities. Supplementation of additional lactococcal strains/culture was reported in the Polish factories where samples BA6 and BB4 were collected from (and group II and III RBPs were identified) as well as the Swedish factory where AO whey was collected (and AO-RBP1, subgroup VA, was identified).

In addition, to confirm and expand upon previously described links between RBP type and the *cwps*-type strains they have the capacity to infect, we also investigated the specificity of several refined and novel RBP types. Attempts were made to correlate these novel RBP groups to a particular *cwps* genotype of potential host strains through fluorescent binding assays. This was successful for three (groups VI, VIII and X) of the seven RBP groups. The binding affinity of RBP groups IV (^His^GFP-RBP_AD1-2_), VII (^His^GFP-RBP_AE3-3_ and ^His^GFP-RBP_BB4-4_) and IX (^His^GFP-RBP_CA-5_) remains unknown as representative ^His^GFP-RBPs of these groups did not appear to bind to any of the CWPS-type strains tested with particularly high affinity. This may be due to poor functioning of the heterologously produced ^His^GFP-RBP fusion proteins or perhaps because these RBPs recognize CWPSs that were not included in this study (either CWPS types not yet identified or defined, or ones not included in our strain collection, such as C_8_-subtype strains). In addition, attempts were not made to determine the binding specificity of RBP group XI, as a representative RBP of this group was not identified within the phageome assemblies. It is expected that as more lactococcal phages and strains are isolated, the RBP grouping system (and links between RBP groups and host CWPS types) will need to be further updated and (likely) expanded.

Along with the phageomic analysis outlined above, the PhRACS approach was used here to exploit the specific phage RBP-host cell surface receptor interactions facilitating the isolation of phage–host combinations from factory-derived whey samples and a mesophilic undefined starter culture. Notably, this method had previously been applied to the successful isolation of a phage–host combination for BB4-RBP2 (group III), resulting in the isolation of a low abundance lactococcal strain (harbouring a B-type *cwps* cluster) and infecting phage (BB4-SC34) with a 100% identical RBP to BB4-RBP2 [23]. In the current study, the use of this method led to the isolation of novel skunaviruses AO-4 and BQ-1 (identified through Sanger sequencing of the RBP gene) capable of infecting C_6_ and C_3_ strains, respectively. Notably, skunaviruses capable of infecting C_6_-type strains had not been described previously (although phage genomes with similar RBP sequences have been deposited in GenBank, the corresponding host *cwps* of these phages was not described [69]).

It is important to note that the ^His^GFP-RBP fusions were expressed only as a single RBP, whereas the complete baseplate of *Skunavirus* p2 is known to consist of a Dit (distal tail protein) hexameric ring, with an RBP trimer attached to each Dit monomer [24]. Structurally, however, all assessed phages were found to possess an attachment to the Dit similar to that of phages p2 [24] and 1358 [70], implying that their RBPs undergo a rotation to be activated and point to the receptor at the bacterial surface. In addition to the RBP, additional carbohydrate-binding modules (CBMs) may be present in auxiliary host cell binding proteins such as the neck passage structure, major tail extension protein (TpeX) and ‘evolved’ Dit [31]. Therefore, the addition of multiple RBP trimers and auxiliary CBMs may impact the phage–host interaction during adsorption, and this study describes only the most probable host of skunaviruses possessing a particular RBP. Despite these limitations, by refining and expanding the RBP groups, it is possible through an analysis of RBP sequences within a population to predict potential hosts of phages present. With a better understanding of the phage–host interactions occurring within these complex microbial communities, it would then be possible to improve starter culture rotation strategies based on both the abundant and emerging phage populations within a fermentation environment. Importantly, this study further demonstrates the specific nature of the interactions of *Skunavirus* RBPs and their lactococcal host CWPS and even possibly highlights a reasonable demand for further investigations into the RBP-CWPS interactions of other Gram-positive ovococcoid bacteria.

## Conclusions

By profiling phage–host interactions occurring in various dairy fermentation factories employing mesophilic undefined starter cultures (primarily composed of lactococcal strains), it was possible to define the intricate and specific phage–host binding interactions that occur between skunaviruses and their cognate hosts. Through sequence analysis and structural predictions, the previous RBP grouping system (groups I–V) has been refined and expanded to now encompass 11 groups (designated group I through to XI) and several subgroups (in the case of groups I, V and VII). Availing of His-tagged GFP-RBP fusion proteins, we successfully demonstrated binding associations between eight RBP (sub)groups and the CWPS type of the corresponding host(s) of these phages. By establishing the specific interactions between skunaviruses and their hosts, more robust and effective starter culture rotation strategies may be applied to reduce the risk of phage infection in food fermentations. In addition, this study demonstrates the increasing genetic and structural diversity exhibited by both host surface-exposed receptors and phage-encoded anti-receptors through the co-evolution of bacteria and their infecting phages.

## Supplementary material

10.1099/mgen.0.001395 Uncited Supplementary Material 1.

## Abbreviations

CBMs: carbohydrate-binding modules
CWPS: cell wall polysaccharide
FU: fluorescence unit
LAB: lactic acid bacteria
PDB: protein database
PhRACS: phage RBP-activated cell sorting
RBP: receptor-binding protein
