# Supplementary material for: Lactococcal phage–host profiling through binding studies between cell wall polysaccharide types and Skunavirus receptor-binding proteins
Source: Microb Genom. 2025 Apr 28;11(4):001395. doi: 10.1099/mgen.0.001395 (PMC12282274; doi:10.1099/mgen.0.001395)
Supplement: Uncited Supplementary Material 1. [file mgen-11-01395-s001.pdf]

Supplementary Table S1. Phageome-derived RBP classification and their GenBank accession numbers

RBP	Length	Head domain	RBP Group	Subgroup	Accession Number
AD1-RBP3	269	p2 group 1	I	A	PQ639349
AD1-RBP5	269	p2 group 1	I	A	PQ639351
AE3-RBP1	269	p2 group 1	I	A	PQ639352
AE3-RBP2	269	p2 group 1	I	A	PQ639353
AI3-RBP1	269	p2 group 1	I	A	PQ639358
AI3-RBP5	269	p2 group 1	I	A	PQ639367
AN3-RBP1	269	p2 group 1	I	A	PQ639361
AN3-RBP2	269	p2 group 1	I	A	PQ639362
AN4-RBP1	269	p2 group 1	I	A	PQ639395
AO-RBP2	269	p2 group 1	I	A	PQ639397
AO-RBP5	241	p2 group 1	I	A	PQ639400
AV6-RBP1	269	p2 group 1	I	A	PQ639401
BC9-RBP1	269	p2 group 1	I	A	PQ639368
BC9-RBP3	269	p2 group 1	I	A	PQ639370
BH1-RBP2	269	p2 group 1	I	A	PQ639414
BP-RBP1	269	p2 group 1	I	A	PQ639415
BQ-RBP4	269	p2 group 1	I	A	PQ639375
BQ-RBP5	269	p2 group 1	I	A	PQ639376
BR-RBP1	241	p2 group 1	I	A	PQ639416
BV-RBP1	269	p2 group 1	I	A	PQ639379
BV-RBP8	269	p2 group 1	I	A	PQ639386
BZ-RBP2	269	p2 group 1	I	A	PQ639419
BZ-RBP4	269	p2 group 1	I	A	PQ639421
CA-RBP2	269	p2 group 1	I	A	PQ639388
CA-RBP8	269	p2 group 1	I	A	PQ639394
CD-RBP1	269	p2 group 1	I	A	PQ639422
V1-RBP1	269	p2 group 1	I	A	PQ639423
V2-RBP3	269	p2 group 1	I	A	PQ639426
AD1-RBP1	269	p2 group 1	I	B	PQ639347
AD1-RBP4	269	p2 group 1	I	B	PQ639350
AF3-RBP1	269	p2 group 1	I	B	PQ639427
AI3-RBP2	269	p2 group 1	I	B	PQ639359
AO-RBP3	269	p2 group 1	I	B	PQ639398
BA6-RBP3	269	p2 group 1	I	B	PQ639404
BC9-RBP2	269	p2 group 1	I	B	PQ639369
BD-RBP2	241	p2 group 1	I	B	PQ639410
BE9-RBP1	269	p2 group 1	I	B	PQ639411
BQ-RBP6	269	p2 group 1	I	B	PQ639377
BQ-RBP7	269	p2 group 1	I	B	PQ639378
BT-RBP1	269	p2 group 1	I	B	PQ639417
BV-RBP5	269	p2 group 1	I	B	PQ639383
BV-RBP6	269	p2 group 1	I	B	PQ639384
BZ-RBP3	269	p2 group 1	I	B	PQ639420
CA-RBP4	269	p2 group 1	I	B	PQ639390
CA-RBP6	269	p2 group 1	I	B	PQ639392

V2-RBP1	269	p2 group 1	I	B	PQ639424
BQ-RBP2	274	p2 group 1	I	C	PQ639373
BV-RBP3	275	p2 group 1	I	C	PQ639381
CA-RBP1	275	p2 group 1	I	C	PQ639387
V2-RBP2	257	p2 group 1	I	D	PQ639425
BA6-RBP1	272	bIL70 group 3	II		PQ639402
BA6-RBP2	274	Tuc2009-like group 4	III		PQ639403
BB4-RBP2	274	Tuc2009-like group 4	III		PQ639406
BV-RBP7	274	Tuc2009-like group 4	III		PQ639385
BZ-RBP1	274	Tuc2009-like group 4	III		PQ639418
CA-RBP3	274	Tuc2009-like group 4	III		PQ639389
AD1-RBP2	273	p2-like group 2	IV		PQ639348
BV-RBP4	269	p2-like group 2	IX		PQ639382
CA-RBP5	269	p2-like group 2	IX		PQ639391
AO-RBP1	275	Phage T4 gp12 group 5	V	A	PQ639396
AE3-RBP4	272	p2-like group 2	VI		PQ639355
AE3-RBP5	272	p2-like group 2	VI		PQ639356
AI3-RBP3	273	p2-like group 2	VI		PQ639360
AN3-RBP3	273	p2-like group 2	VI		PQ639363
AN3-RBP5	273	p2-like group 2	VI		PQ639365
BB4-RBP1	273	p2-like group 2	VI		PQ639405
BB4-RBP3	273	p2-like group 2	VI		PQ639407
BC9-RBP4	264	p2-like group 2	VI		PQ639371
AE3-RBP3	348	p2-like group 2	VII	A	PQ639354
AF9-RBP1	316	p2-like group 2	VII	A	PQ639357
AI3-RBP4	317	p2-like group 2	VII	A	PQ639366
AN3-RBP4	348	p2-like group 2	VII	A	PQ639364
BB4-RBP4	348	p2-like group 2	VII	A	PQ639408
AO-RBP4	267	p2-like group 2	VIII		PQ639399
BQ-RBP3	267	p2-like group 2	VIII		PQ639374
BV-RBP2	267	p2-like group 2	VIII		PQ639380
BD-RBP1	285	p2-like group 2	X		PQ639409
BE9-RBP2	285	p2-like group 2	X		PQ639412
BH1-RBP1	285	p2-like group 2	X		PQ639413
BQ-RBP1	285	p2-like group 2	X		PQ639372
CA-RBP7	285	p2-like group 2	X		PQ639393

Supplementary Table S2. Oligonucleotides used in this study.

Targeted RBP(s)	Targeted *orf*	Oligonucleotide	Sequence (5' -3')
AD1-RBP2	$orf_{AD1\text{-}RBP2}$	AD1-2_F	**TCAGCAAGGGCTGAGG** ATGACAATTAAAAGCTTCACATTTTTC
		AD1-2_R	**TCAGCGGAAGCTGAGG** TTATGCTGGGAATGAATCACCAGTG
AE3-RBP3, BB4-RBP4	$orf_{AE3\text{-}RBP3}$, $orf_{\text{BB4-RBP4}}$	AE3-3_F	**TCAGCAAGGGCTGAGG** ATGACAATTAAAAACTTCACATTTTTC
		AE3-3_R	**TCAGCGGAAGCTGAGG**TTATTTATGCTTGCTAGCTTGTC
AO-RBP1	$orf_{AO\text{-}RBP1}$	AO1_F	**TCAGCAAGGGCTGAGG**ATGACAATTAAAAATTTTACATTTTTCAGTCC
		AO1_R	**TCAGCGGAAGCTGAGG**CTAATTTCTATAAAGTTTTACAATC
AO-RBP2,_AD1-RBP5	$orf_{AO\text{-}RBP2}$, $orf_{\text{BB4AD1-RBP5}}$	AO2AD15_F	**TCAGCAAGGGCTGAGG**ATGACAATTAAAAATTTCACG
		AO2AD15_R	**TCAGCGGAAGCTGAGG**TTATTTAATAAAGTAGCTTGCG
AO-RBP4	$orf_{\text{AO-RBP4}}$	AO4_F	**TCAGCAAGGGCTGAGG**ATGACGATTAAAAACTTTACATTTTTC
		AO4_R	**TCAGCGGAAGCTGAGG**CTATTTTTTAGTTGCTATAAATTC
BA6-RBP1	$orf_{\text{BA6-RBP1}}$	BA61_F	**TCAGCAAGGGCTGAGG**ATGACAATTAAAAACTTTACATTCTTTAG
		BA61_R	**TCAGCGGAAGCTGAGG**TTACAATAAAGGAGCTGATAAAGCG
BA6-RBP2	$orf_{\text{BA6-RBP2}}$	BA62_F	**TCAGCAAGGGCTGAGG**ATGACAATTAAAAAATTCACG
		BA62_R	**TCAGCGGAAGCTGAGG**CTAATTTATACGTTTCCAAATATAG
BB4-MG12	$orf_{\text{BB4-MG12}}$	C-type_F	**TCAGCAAGGGCTGAGG**ATGACAATTAAAAACTTTAC
		B-MG12_R	**TCAGCGGAAGCTGAGG**TTATTTTATAAAGTAACTGG
BB4-RBP1	$orf_{\text{BB4-RBP1}}$	BB42_F	**TCAGCAAGGGCTGAGG**ATGACAATTAAAAAATTCACGTT
		BB42_R	**TCAGCGGAAGCTGAGG**CTAATTTATACGTTTCCAAATAT
BB4-RBP2	$orf_{\text{BB4-RBP2}}$	BB4-RBPF*	**TCAGCAAGGGCTGAGG**ATGACAATTAAAAAATTCACGTT
		BB4-RBPR*	**TCAGCGGAAGCTGAGG**CTAATTTATACGTTTCCAAATAT
BQ-RBP1	$orf_{\text{BQ-RBP1}}$	BQ1_F	**TCAGCAAGGGCTGAGG**ATGACAATTAAAAACTTTACATTCTTTAG
		BQ1_R	**TCAGCGGAAGCTGAGG**TCAATATTCTAATACGTGCTGTAG
CA_RBP5	$orf_{\text{CA-RBP5}}$	CA5_F	**TCAGCAAGGGCTGAGG** ATGACAATTAAAAACTTTACATTTTTC
		CA5_F	**TCAGCGGAAGCTGAGG**TTATAAAGTTTTTGTCGCAAATAC
V2-RBP2	$orf_{\text{V2-RBP2}}$	C-type_F	**TCAGCAAGGGCTGAGG**ATGACAATTAAAAACTTTAC
		V2-2_R	**TCAGCGGAAGCTGAGG**TTATTTGATGAAATACATGGC

*Primers first described in previous study [1].

Supplementary Table S3. Absolute and filtered reads of sequenced whey virome samples (BioSample accession numbers can be found in Table 1).

Virome	Sequencing pair-end reads (bp)	Quality filtered reads (bp)
T1	1,133,867	1,037,855
BH1	2,568,678	2,221,469
BR	6,214,791	6,060,432
BT	3,547,612	2,996,672
AD1	2,551,593	2,077,891
AF3	2,315,752	1,912,768
AF9	3,409,093	2,891,303
BQ	5,063,789	4,927,315
BA6	3,717,233	3,460,088
BB4	2,614,285	2,521,910
BZ	6,194,746	6,031,224
AO	5,660,413	4,315,150
V1	1,271,906	1,196,962
V2	1,811,806	1,618,823
AE3	4,217,248	3,945,684
AI3	2,463,249	2,341,785
AM2	108,056	33,317
AN3	1,263,488	1,068,294
AN4	3,292,791	3,131,523
AV6	3,224,986	2,895,954
BC9	4,802,406	4,506,953
BD	5,123,477	4,697,018
BE9	5,456,904	5,056,351
BV	5,059,960	4,832,490
CA	7,704,661	7,436,758
CD	6,824,593	6,609,464
BP	6,013,683	5,434,002

Supplementary Table S4. Whey virome reads taxonomic profiling.

Genera	T1	BH1	BR	BT	AD1	AF3	AF9	BQ	BA6	BB4	BZ	AO	BS	V1	V2	AE3	AI3	AM2	AN3	AN4	AV6	BC9	BD	BE9	BV	CA	CD	BP
Eukaryota-Fungi_Cantharellus	0%	0%	0%	0%	0%	0%	0%	0%	0%	0%	0%	0%	0%	0%	0%	0%	0%	0%	0%	0%	2%	0%	0%	0%	0%	0%	10%	0%
Eukaryota-Fungi_Echinosphaeria	0%	0%	0%	0%	0%	0%	0%	0%	0%	0%	0%	0%	0%	0%	0%	0%	0%	2%	2%	0%	0%	0%	0%	0%	0%	0%	0%	0%
Eukaryota-Fungi_Geoglossum	0%	0%	0%	0%	0%	0%	0%	0%	0%	0%	0%	0%	0%	0%	0%	0%	0%	1%	1%	0%	6%	0%	0%	0%	0%	0%	27%	0%
Eukaryota-Protist_Rostrostelium	0%	0%	0%	0%	0%	0%	0%	0%	0%	0%	0%	0%	0%	0%	0%	0%	0%	0%	0%	0%	0%	2%	0%	0%	0%	0%	0%	0%
Prokaryote_Achromobacter	0%	0%	0%	0%	0%	0%	0%	0%	0%	0%	0%	0%	0%	0%	0%	0%	0%	1%	0%	0%	0%	1%	0%	0%	0%	0%	0%	0%
Prokaryote_Acinetobacter	0%	0%	0%	0%	0%	0%	1%	0%	0%	0%	0%	0%	0%	0%	0%	0%	0%	0%	0%	0%	0%	0%	0%	0%	0%	0%	0%	0%
Prokaryote_Agrobacterium	0%	0%	0%	0%	0%	0%	0%	0%	0%	0%	0%	0%	0%	0%	0%	0%	0%	0%	0%	0%	0%	3%	0%	0%	0%	0%	0%	0%
Prokaryote_Bacillus	1%	0%	1%	0%	0%	0%	0%	0%	0%	0%	0%	0%	0%	0%	0%	4%	2%	31%	34%	0%	0%	0%	0%	0%	0%	0%	0%	0%
Prokaryote_Bacteroides	0%	0%	0%	0%	0%	0%	0%	0%	0%	0%	0%	0%	0%	0%	0%	0%	0%	0%	0%	0%	0%	0%	0%	0%	0%	0%	0%	1%
Prokaryote_Confluentibacter	0%	0%	0%	0%	0%	0%	0%	0%	0%	0%	0%	0%	0%	0%	0%	0%	0%	0%	0%	0%	0%	0%	0%	0%	0%	0%	1%	0%
Prokaryote_Cutibacterium	0%	0%	0%	0%	0%	0%	0%	0%	0%	0%	0%	0%	0%	0%	0%	0%	0%	2%	1%	0%	0%	0%	0%	0%	0%	0%	0%	0%
Prokaryote_Enterococcus	0%	0%	0%	0%	1%	1%	0%	0%	1%	0%	0%	0%	0%	0%	0%	0%	0%	0%	0%	0%	0%	0%	0%	0%	0%	0%	0%	0%
Prokaryote_Faecalibacterium	0%	0%	0%	0%	0%	0%	0%	0%	0%	0%	0%	0%	0%	0%	0%	0%	0%	0%	0%	0%	0%	0%	0%	0%	0%	0%	0%	1%
Prokaryote_Flavobacterium	0%	0%	0%	0%	0%	0%	0%	0%	0%	0%	0%	0%	0%	0%	0%	0%	0%	0%	0%	0%	1%	0%	0%	0%	0%	0%	4%	0%
Prokaryote_Herbaspirillum	0%	0%	0%	0%	0%	0%	0%	0%	0%	0%	0%	0%	0%	0%	0%	0%	0%	0%	0%	0%	0%	0%	0%	0%	0%	0%	1%	0%
Prokaryote_Klebsiella	0%	0%	0%	0%	0%	3%	0%	0%	0%	0%	0%	0%	0%	0%	0%	0%	0%	8%	3%	0%	0%	0%	0%	0%	0%	0%	0%	0%
Prokaryote_Lactobacillus	0%	0%	0%	0%	1%	1%	0%	0%	1%	0%	0%	0%	0%	0%	0%	0%	0%	0%	0%	0%	0%	0%	0%	0%	0%	0%	0%	0%
Prokaryote_Lactococcus	13%	32%	12%	6%	95%	1%	0%	3%	84%	1%	0%	4%	4%	4%	4%	12%	12%	34%	46%	3%	4%	1%	0%	2%	0%	0%	0%	5%
Prokaryote_Lelliottia	0%	0%	0%	0%	0%	0%	0%	0%	0%	0%	0%	1%	0%	0%	0%	0%	0%	0%	0%	0%	0%	26%	0%	0%	0%	0%	0%	0%
Prokaryote_Microbacterium	0%	0%	0%	0%	0%	0%	0%	0%	0%	0%	0%	0%	0%	0%	0%	0%	0%	1%	0%	0%	0%	1%	0%	0%	0%	0%	0%	0%
Prokaryote_Mycolicibacterium	0%	0%	0%	0%	0%	0%	0%	0%	0%	0%	0%	0%	0%	0%	0%	0%	0%	2%	0%	0%	0%	0%	0%	0%	0%	0%	0%	0%
Prokaryote_Ochrobactrum	0%	0%	0%	0%	0%	0%	0%	0%	0%	0%	0%	0%	0%	0%	0%	0%	0%	3%	0%	0%	0%	0%	0%	0%	0%	0%	0%	0%
Prokaryote_Pantoea	0%	0%	0%	0%	0%	0%	0%	0%	0%	0%	0%	0%	0%	0%	0%	0%	0%	0%	0%	0%	0%	1%	0%	0%	0%	0%	0%	0%
Prokaryote_Paraburkholderia	0%	0%	0%	0%	0%	0%	0%	0%	0%	0%	0%	0%	0%	0%	0%	0%	0%	0%	0%	0%	0%	0%	0%	0%	0%	0%	1%	0%
Prokaryote_Pseudomonas	0%	0%	0%	0%	0%	0%	0%	0%	0%	0%	0%	0%	0%	0%	0%	0%	0%	0%	0%	0%	2%	0%	0%	0%	0%	0%	10%	0%
Prokaryote_Rahnella	0%	0%	0%	0%	0%	0%	0%	0%	0%	0%	0%	0%	0%	0%	0%	0%	0%	0%	0%	0%	0%	4%	0%	0%	0%	0%	0%	0%
Prokaryote_Rhizobium	0%	0%	0%	0%	0%	0%	0%	0%	0%	0%	0%	1%	0%	0%	0%	0%	0%	0%	0%	0%	0%	8%	0%	0%	0%	0%	0%	0%
Prokaryote_Sporosarcina	0%	0%	0%	0%	0%	0%	0%	0%	0%	0%	0%	0%	0%	0%	0%	0%	0%	0%	0%	0%	0%	0%	0%	0%	0%	0%	1%	0%
Prokaryote_Stenotrophomonas	0%	0%	0%	0%	0%	0%	0%	0%	0%	0%	0%	0%	0%	0%	0%	0%	0%	10%	2%	0%	0%	16%	0%	0%	0%	0%	0%	0%
Prokaryote_Streptococcus	0%	0%	0%	0%	1%	14%	10%	0%	1%	0%	0%	0%	0%	0%	0%	0%	0%	0%	0%	0%	0%	0%	0%	0%	0%	0%	2%	0%
Prokaryote_Xanthomonas	0%	0%	0%	0%	0%	0%	0%	0%	0%	0%	0%	0%	0%	0%	0%	0%	0%	0%	0%	0%	0%	1%	0%	0%	0%	0%	0%	0%
Virus_Demerecviridae	0%	0%	0%	0%	0%	0%	0%	0%	0%	0%	0%	0%	0%	0%	0%	0%	0%	0%	0%	0%	2%	0%	0%	0%	0%	0%	11%	0%
Virus_Myoviridae	0%	0%	0%	0%	0%	0%	0%	0%	0%	0%	0%	0%	0%	0%	0%	0%	0%	0%	0%	0%	5%	0%	0%	0%	0%	0%	25%	0%
Virus_Podoviridae	0%	0%	0%	0%	0%	0%	0%	2%	0%	0%	0%	0%	0%	0%	0%	0%	0%	0%	0%	0%	2%	0%	0%	0%	0%	0%	9%	0%
Virus_Poxviridae	0%	0%	0%	0%	0%	0%	0%	0%	0%	0%	0%	0%	0%	0%	0%	0%	0%	2%	1%	0%	0%	0%	0%	0%	0%	0%	0%	0%
Virus_Siphoviridae	86%	68%	87%	94%	2%	81%	89%	95%	13%	99%	100%	94%	96%	96%	96%	84%	86%	3%	10%	97%	76%	36%	100%	98%	100%	100%	0%	93%

Supplementary Table S5. Relative abundance of *Skunavirus* RBP groups present in whey virome samples. The total number of reads, in a given sample, that mapped against any of the RBP groups is indicated in red.

Whey Viromes:			AD1	BP	V1	V2	AE3	AI3	AM2*†	AN3	AN4	AV6	BC9	BD	BE9	BV	CA	CD*	AF3*	AF9*	BQ	BA6	BB4	BZ	T1*	BH1	BR*	BT	AO
(Total reads mapped)			6095	143019	37885	23440	120063	58212	54	2979	106815	11911	66043	225378	225432	179810	264538	63	70	385	240842	25955	138646	240708	0	1282	50	77519	128418
RBP groups	Group IA	(CWPS type C)	92.2	99.7	96.7	14.5	93.2	98.7	0.0	22.7	99.9	97.1	97.7	59.5	3.5	33.2	23.7	58.5	33.9	7.1	0.4	0.3	0.0	2.8	0.0	91.7	100.0	93.0	51.8
	Group IB		1.6	0.2	3.3	85.5	4.1	0.0	0.0	0.6	0.0	0.0	1.7	3.4	1.6	1.2	1.9	0.0	16.1	0.0	2.2	2.3	0.0	0.1	0.0	0.0	0.0	6.3	17.8
	Group IC		0.0	0.0	0.0	0.0	0.0	0.0	0.0	0.0	0.0	0.0	0.0	0.0	0.0	9.2	26.7	0.0	0.0	0.0	27.9	0.3	0.0	0.0	0.0	0.0	0.0	0.1	3.6
	Group ID		0.0	0.0	0.0	0.0	0.0	0.0	0.0	0.0	0.0	0.0	0.0	0.0	0.0	0.0	0.0	0.0	0.0	0.0	0.0	0.0	0.0	0.0	0.0	0.0	0.0	0.0	0.0
	Group II	(CWPS type B)	0.0	0.0	0.0	0.0	0.1	0.0	0.0	2.7	0.0	0.0	0.0	0.0	0.0	0.0	0.0	0.0	0.0	0.0	0.0	87.8	0.0	0.0	0.0	0.0	0.0	0.0	0.0
	Group III	(CWPS type B & C)	0.0	0.0	0.0	0.0	0.0	0.0	0.0	2.0	0.0	0.1	0.0	0.0	0.0	0.7	0.1	0.0	6.3	5.0	0.0	9.1	1.1	97.1	0.0	0.0	0.0	0.0	0.0
	Group IV	(CWPS type U)	6.2	0.0	0.0	0.0	0.0	0.0	0.0	0.0	0.0	0.0	0.0	0.0	0.0	0.0	0.0	0.0	0.0	0.0	0.0	0.0	0.0	0.0	0.0	0.0	0.0	0.0	0.0
	Group VA	(CWPS type A)	0.0	0.0	0.0	0.0	0.0	0.0	0.0	0.0	0.0	0.0	0.0	0.0	0.0	0.0	0.0	0.0	0.0	0.0	0.0	0.0	0.0	0.0	0.0	0.0	0.0	0.0	11.2
	Group VB	(CWPS type A)	0.0	0.0	0.0	0.0	0.0	0.0	0.0	0.0	0.0	0.0	0.0	0.0	0.0	0.0	0.6	0.0	0.0	0.0	0.0	0.0	0.0	0.0	0.0	0.0	0.0	0.0	0.0
	Group VI	(CWPS type D)	0.0	0.0	0.0	0.0	0.3	0.2	0.0	10.1	0.0	0.3	0.3	0.0	0.0	0.0	0.0	0.0	0.0	7.9	0.0	0.0	14.2	0.0	0.0	0.0	0.0	0.0	0.0
	Group VIIA	(CWPS type U)	0.0	0.0	0.0	0.0	2.2	1.0	0.0	62.0	0.1	2.5	0.0	0.0	0.0	0.0	0.0	0.0	43.6	80.0	0.0	0.1	84.7	0.0	0.0	0.0	0.0	0.0	0.0
	Group VIIB	(CWPS type U)	0.0	0.0	0.0	0.0	0.0	0.0	0.0	0.0	0.0	0.0	0.0	0.0	0.0	0.0	0.0	0.0	0.0	0.0	0.0	0.0	0.0	0.0	0.0	0.0	0.0	0.0	0.0
	Group VIII	(CWPS type C_6)	0.0	0.0	0.0	0.0	0.0	0.0	0.0	0.0	0.0	0.0	0.3	0.0	0.0	55.1	43.2	41.5	0.0	0.0	0.3	0.0	0.0	0.0	0.0	0.0	0.0	0.4	15.0
	Group IX	(CWPS type U)	0.0	0.0	0.0	0.0	0.0	0.0	100.0	0.0	0.0	0.0	0.0	0.0	0.0	0.5	1.2	0.0	0.0	0.0	0.0	0.0	0.0	0.0	0.0	0.0	0.0	0.0	0.7
	Group X	(CWPS type C_3)	0.0	0.0	0.0	0.0	0.0	0.0	0.0	0.0	0.0	0.0	0.0	37.2	94.9	0.0	2.7	0.0	0.0	0.0	69.1	0.0	0.0	0.0	0.0	8.3	0.0	0.1	0.0
	Group XI	(CWPS type U)	0.0	0.0	0.0	0.0	0.0	0.0	0.0	0.0	0.0	0.0	0.0	0.0	0.0	0.0	0.0	0.0	0.0	0.0	0.0	0.0	0.0	0.0	0.0	0.0	0.0	0.0	0.0
	None		0.0	0.0	0.0	0.0	0.0	0.0	0.0	0.0	0.0	0.0	0.0	0.0	0.0	0.0	0.0	0.0	0.0	0.0	0.0	0.0	0.0	0.0	100.0	0.0	0.0	0.0	0.0

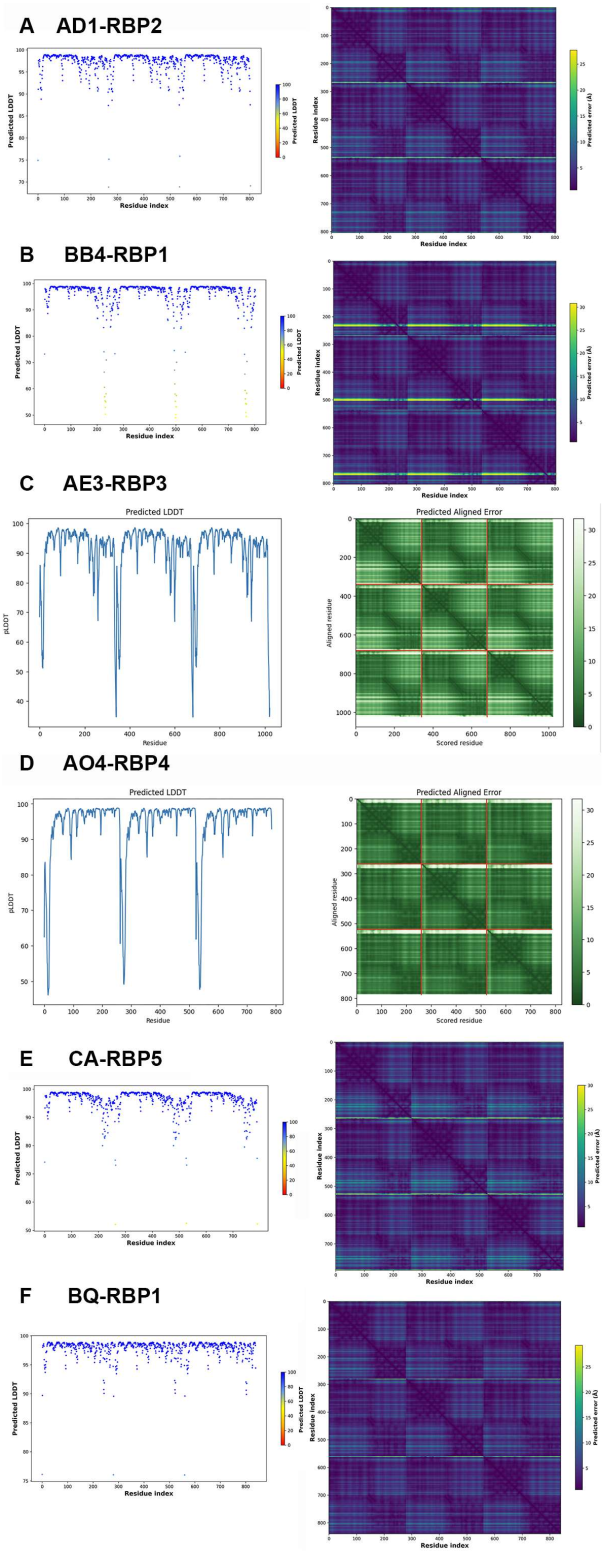

Supplementary Figure S1. pLDDT values and predicted aligned errors (PAE) plots of predicted structures. The pLDDT plots (left) and PAE plots (right) correspond to RBP trimers; the numbering is that of the main RBPs Figure.

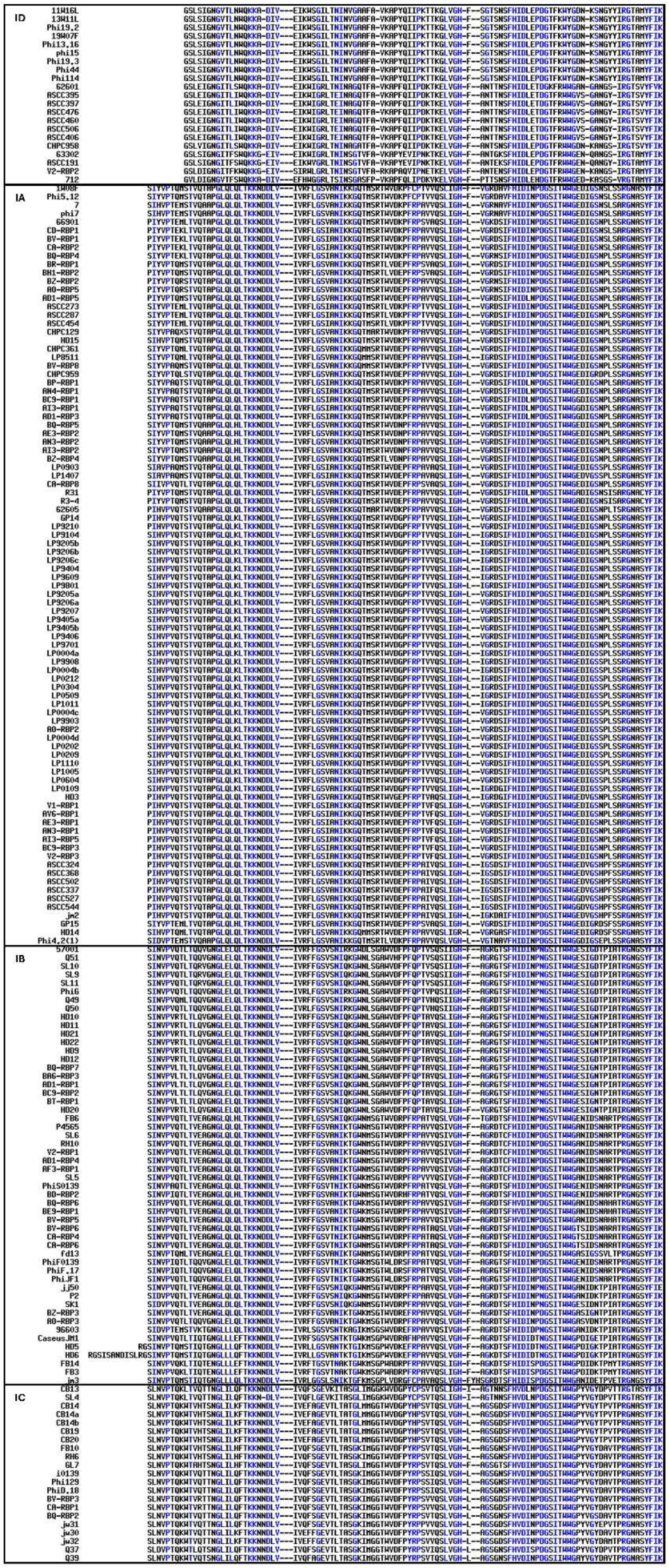

Supplementary Figure S2. Alignment of group I RBP head domains.

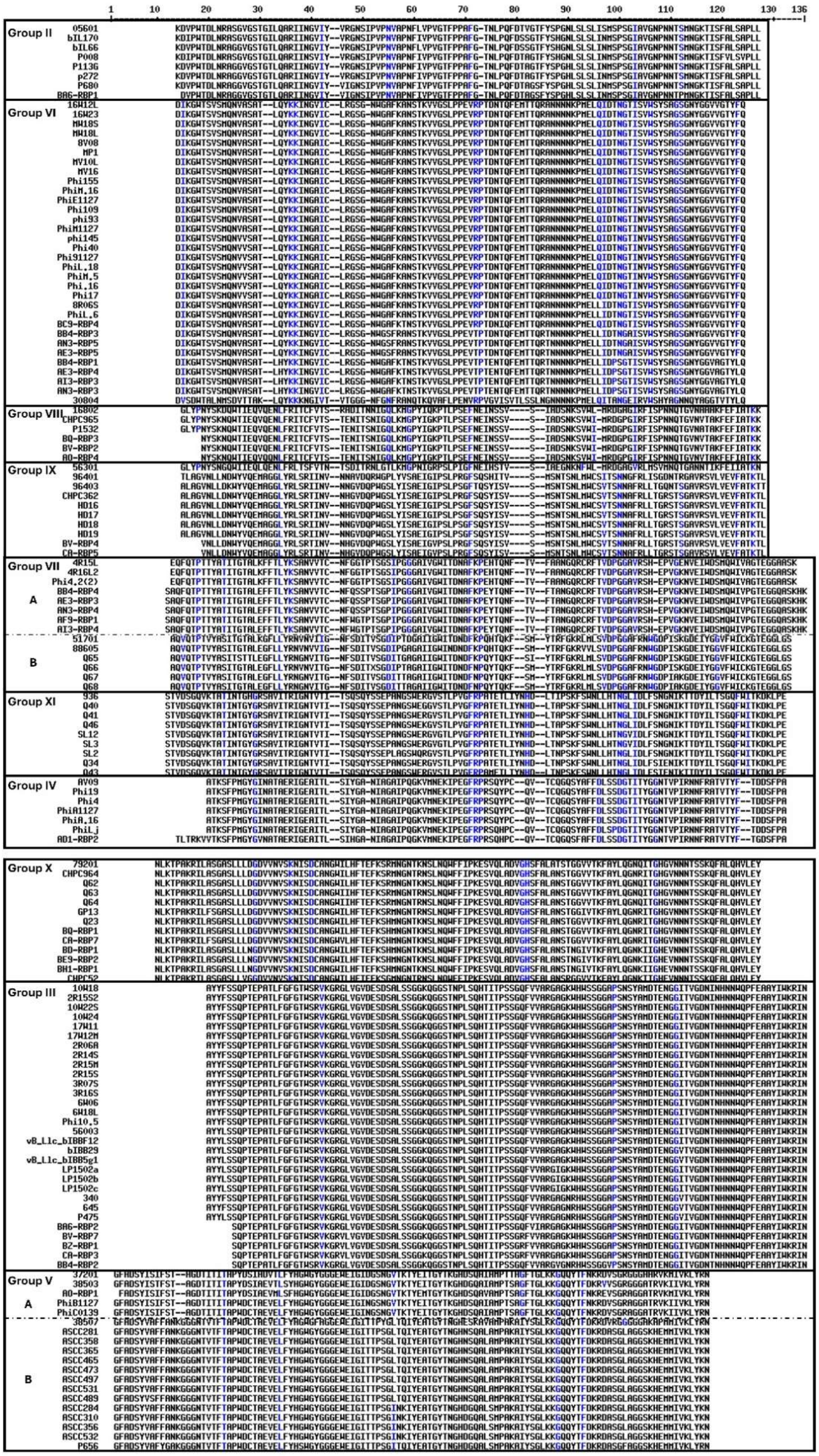

Supplementary Figure S3. Alignment of RBP groups II-XI head domains.

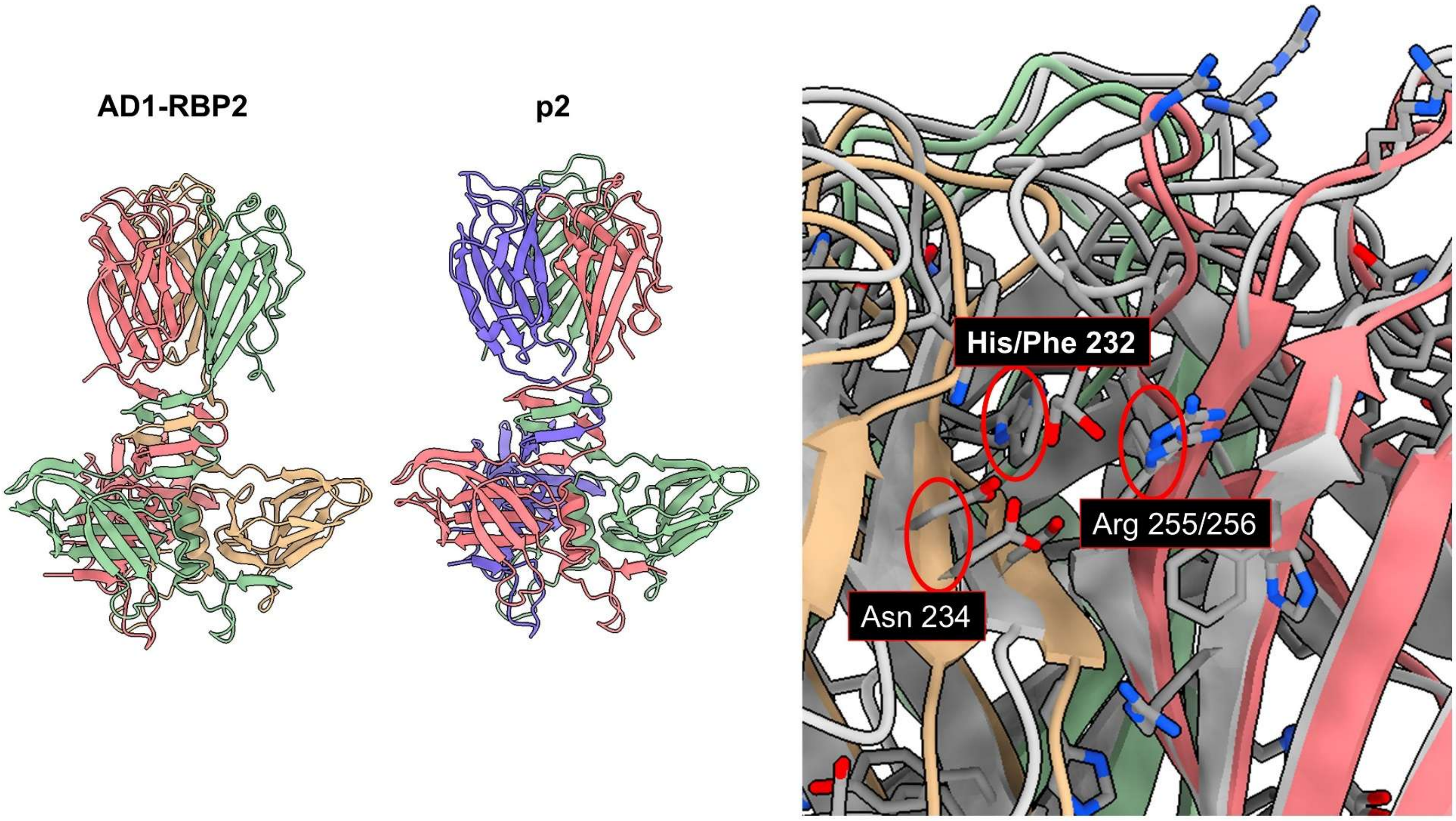

Supplementary Figure S4. Group IV representative AD1-RBP2 shares two residues (Asn 234 and Arg 255/256) located in the binding crevice of skunavirus p2.

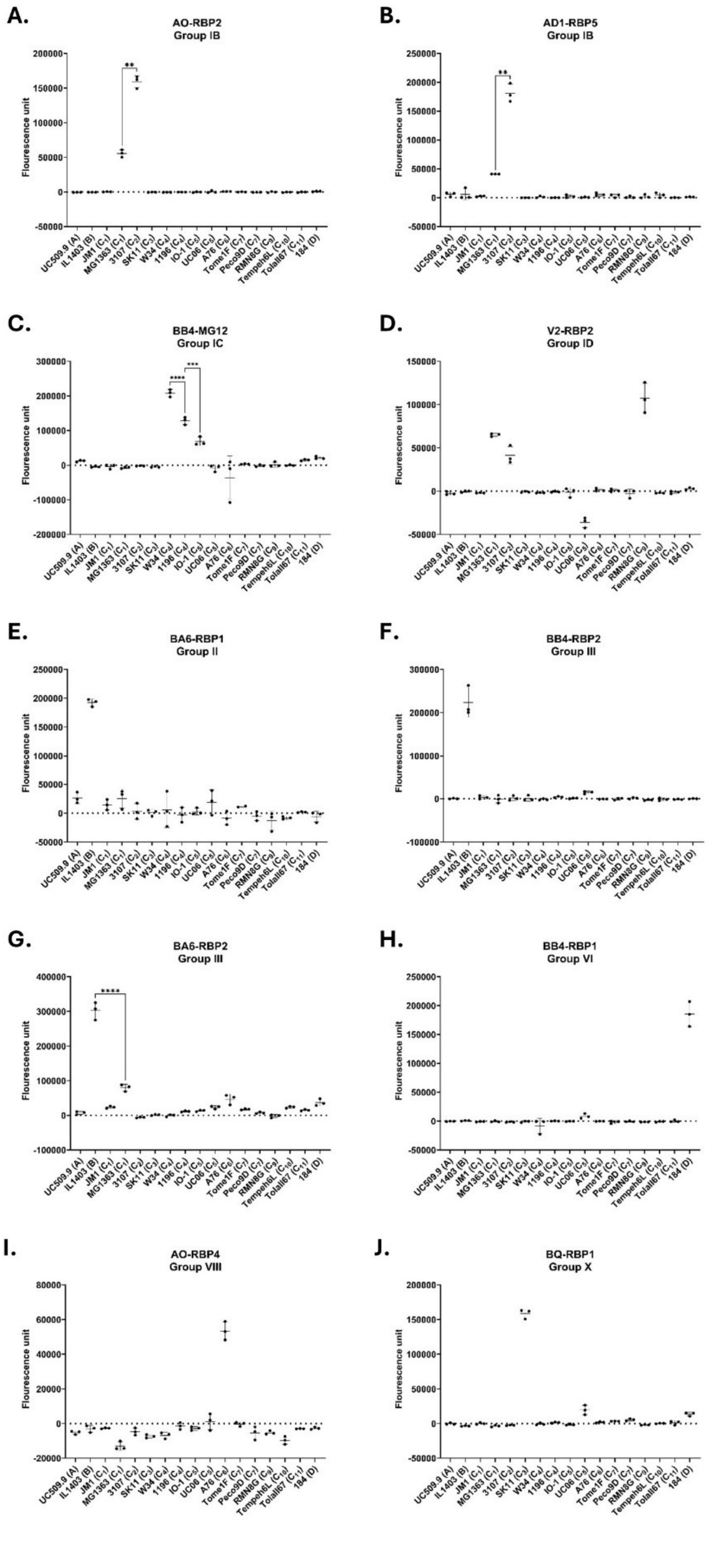

Supplementary Figure S5. Fluorescence-based quantification of binding of ten indicated HisGFP-RBPs where it was possible to confirm the binding specificity of each HisGFP-RBPs against 17 representative lactococcal strains encoding various *cwps* genotypes (A, B, C_1-C_7, C_9-C_{11}, and D).

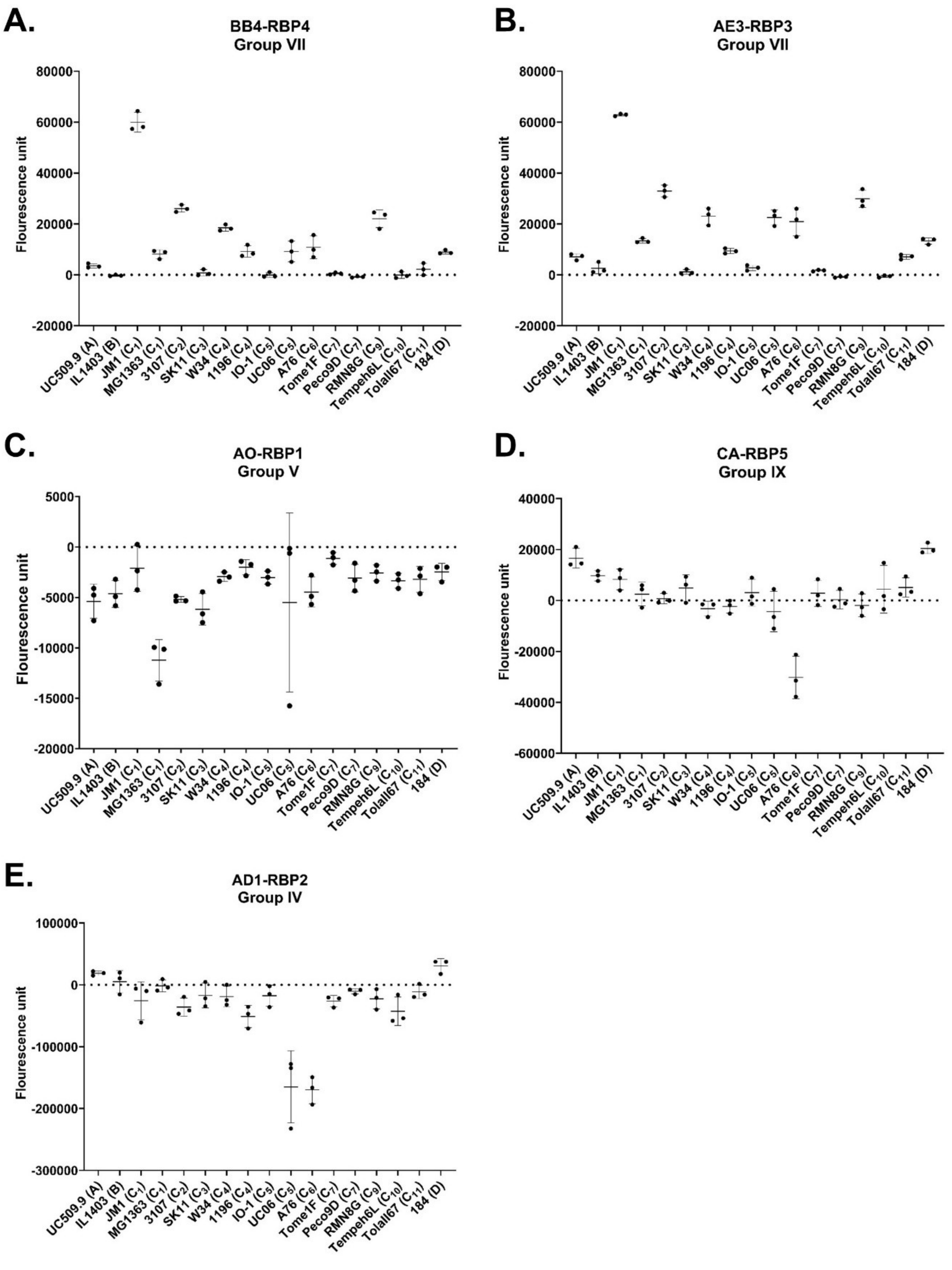

Supplementary Figure S6. Fluorescence-based quantification of binding of five indicated ^{His}GFP-RBPs to 17 representative lactococcal strains encoding various *cwps* genotypes (A, B, C_1-C_7, C_9-C_{11}, and D). It was not possible to establish the binding specificity for these five ^{His}GFP-RBPs (representing groups IV, V, IX and VII) in the confines of this study.

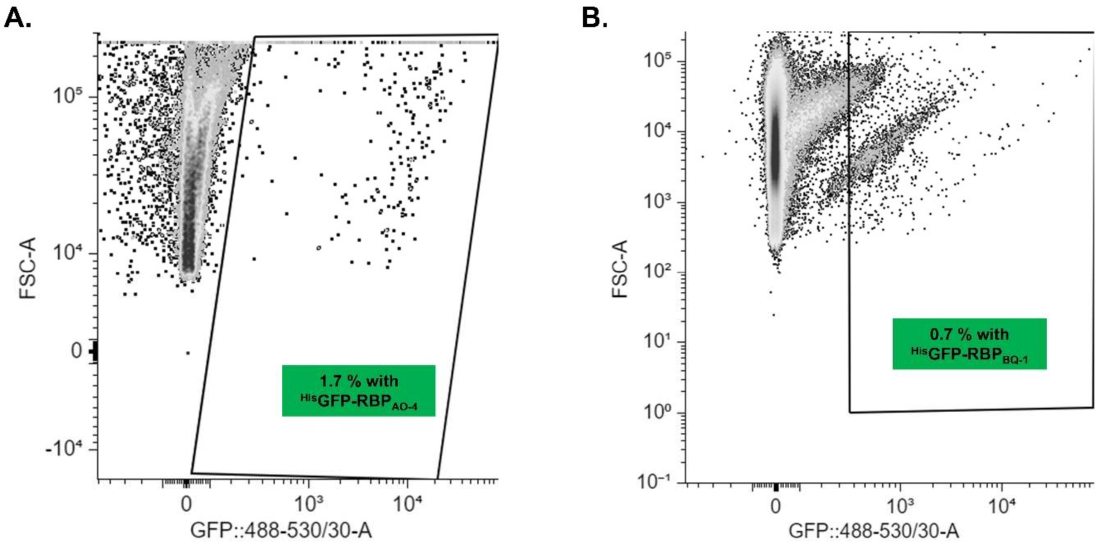

Supplementary Figure S7. Dot analysis of undefined culture M1 labelled with (A) ^{His}GFP-RBP$_{AO-4}$ and (B) ^{His}GFP-RBP$_{BQ-1}$.

A.

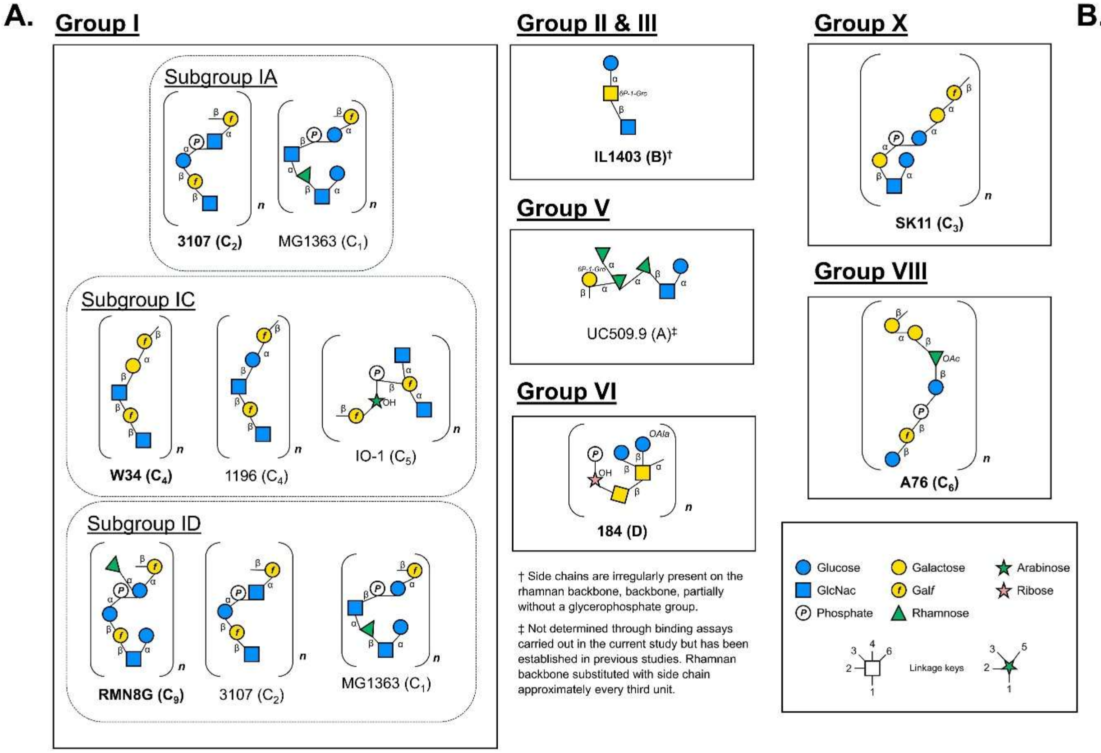

B.

^{His}GFP-RBP	RBP Cluster	Strain & CWPS	Side chain structure
AO-RBP2, AD1-RBP5	IA	3107 (C_2)*	-2-β-Gal*f*-6-α-GlcNAc-*P*-6-α-Glc-3-β-Gal*f*-3-β-GlcNAc-
		MG1363 (C_1)*	-2-β-Gal*f*-6-a-Glc-*P*-6-β-GlcNAc-3-α-Rha-3-β-GlcNAc-<br>|6<br>α-Glc
BB4-MG12	IC	W34 (C_4)*	-6-β-Gal*f*-6-α-Glc-6-β-GlcNAc-3-β-Gal*f*-3-β-GlcNAc-
		1196 (C_4)*	-6-β-Gal*f*-6-α-Gal-6-β-GlcNAc-3-β-Gal*f*-3-β-GlcNAc-
		IO-1 (C_5)*	-2-β-Gal*f*-5-Ara-ol-1-*P*-2-β-Gal*f*-3-α-GlcNAc-<br>|3<br>α-GlcNAc
V2-RBP2	ID	RMN8G (C_9)*	α-Rha<br>|2<br>-2-β-Gal*f*-6-α-Glc-1-*P*-6-α-Glc-3-β-Gal*f*-3-β-GlcNAc-<br>|6<br>α-Glc
		3107 (C_2)*<br>MG1363 (C_1)*	-2-β-Gal*f*-6-α-GlcNAc-*P*-6-α-Glc-3-β-Gal*f*-3-β-GlcNAc-<br>-2-β-Gal*f*-6-a-Glc-*P*-6-β-GlcNAc-3-α-Rha-3-β-GlcNAc-<br>|6<br>α-Glc
BA6-RBP1	II	IL1403 (B)†	β-Glc-4-α-GalNAc(6*P*-1-Gro)-3-β-GlcNAc-
BB4-RBP2	III		
BA6-RBP2			
BB4-RBP1	VI	184 (D)*	β-Glc<br>|3<br>-6-α-GalNAc-3-β-GalNAc-5-Ribitol-1-*P*-<br>|4<br>β-Glc*6OAla*
AE3-RBP3, BB4-RBP4	VIIB	N.D.	
AD1-RBP2	IV	N.D.	
BQ-RBP1	X	SK11 (C_3)*	-6-β-Gal*f*-6-α-Gal-6-α-Glc-*P*-6-α-Gal-3-β-GlcNAc-<br>|6<br>α-Glc
AO-RBP4	VIII	A76 (C_6)*	-6-β-Gal-2-α-Gal-3-β-RhaOAc-4-β-Glc6*P*-6-β-Gal*f*-6-β-Glc-
CA-RBP5	IX	N.D.	
AO-RBP1‡	V	UC509.9 (A)‡	β-Gal(6*P*-1-Gro)-2-α-Rha-2-α-Rha-3-β-GlcNAc-<br>|3 |6<br>α-Rha α-Glc

* Side chains are present as repeating units on the rhamnan backbone.

† Side chains are irregularly present on the rhamnan backbone, backbone, partially without a glycerophosphate group.

‡ Not determined through binding assays carried out in the current study but has been established in previous studies [7, 8]. Rhamnan backbone substituted with side chain approximately every third unit.

Supplementary Figure S8. Symbolic representation of side chain component of lactococcal strains (as determined by previous studies [2–5]) to which a particular ^{His}GFP-RBP was able to bind to (A). The name of the optimal host strain (the strain to which a given ^{His}GFP-RBP consistently binds with the highest affinity) is indicate in bold. Full representation of the surface-exposed side chain component of the CWPS of lactococcal strains representative *Skunavirus* RBPs were capable of binding to (B).

References

1. **Casey E, McDonnell B, White K, Stamou P, Crowley T, *et al.*** Needle in a whey-stack: PhRACS as a discovery tool for unknown phage-host combinations. *mBio* 2022;13:e03334-21. DOI: 10.1128/mbio.03334-21.

2. **Parlindungan E, Sadovskaya I, Vinogradov E, Lugli GA, Ventura M, *et al.*** Novel cell wall polysaccharide genotypes and structures of lactococcal strains isolated from milk and fermented foods. *Int J Food Microbiol* 2024;110840. DOI: 10.1016/j.ijfoodmicro.2024.110840.

3. **Mahony J, Frantzen C, Vinogradov E, Sadovskaya I, Theodorou I, *et al.*** The CWPS Rubik's cube: linking diversity of cell wall polysaccharide structures with the encoded biosynthetic machinery of selected *Lactococcus lactis* strains. *Mol Microbiol* 2020;114:582–596. DOI: 10.1111/mmi.14561.

4. **Chapot-Chartier MP, Vinogradov E, Sadovskaya I, Andre G, Mistou MY, *et al.*** Cell surface of *Lactococcus lactis* is covered by a protective polysaccharide pellicle. *J Biol Chem* 2010;285:10464–10471. DOI: 10.1074/jbc.M109.082958.

5. **Ainsworth S, Sadovskaya I, Vinogradov E, Courtin P, Guerardel Y, *et al.*** Differences in lactococcal cell wall polysaccharide structure are major determining factors in bacteriophage sensitivity. *mBio* 2014;5:e00880-14. DOI: 10.1128/mBio.00880-14.
